# A graphical approach to assess the goodness-of-fit of random-effects linear models when the goal is to measure individual benefits of medical treatments in severely ill patients

**DOI:** 10.1186/s12874-020-01054-3

**Published:** 2020-07-20

**Authors:** Zhiwen Wang, Francisco J. Diaz

**Affiliations:** Department of Biostatistics & Data Science, The University of Kansas Medical Center, Mail Stop 1026, 3901 Rainbow Blvd, Kansas City, KS 66160 USA

**Keywords:** Chronic diseases, Cramer-von Mises discrepancy, Disease severity, Empirical Bayes, Goodness-of-fit, Individual treatment benefits, Normality assumption, Personalized medicine models

## Abstract

**Background:**

Two-dimensional personalized medicine (2-PM) models are tools for measuring individual benefits of medical treatments for chronic diseases which have potential applications in personalized medicine. These models assume normality for the distribution of random effects. It is necessary to examine the appropriateness of this assumption. Here, we propose a graphical approach to assessing the goodness-of-fit of 2-PM models with continuous responses.

**Methods:**

We propose benefit quantile-quantile (BQQ) plots which compare the empirical quantiles of individual benefits from a patient sample predicted through an empirical Bayes (EB) approach versus the quantiles of the theoretical distribution of individual benefits derived from the assumption of normality for the random effects. We examine the performance of the approach by conducting a simulation study that compared 2-PM models with non-normal distributions for the random effects versus models with comparable normal distributions. Cramer-von Mises discrepancies were used to quantify the performance of the approach. The approach was illustrated with data from a clinical trial of imipramine for patients with depression.

**Results:**

Simulations showed that BQQ plots were able to capture deviations from the normality assumption for the random effects and did not show any asymmetric deviations from the *y* = *x* line when the random effects were normally distributed. For the depression data, the points of the BQQ plot were scattered around closely to the *y* = *x* line, without presenting any asymmetric deviations. This implied the adequacy of the normality assumption for the random effects and the goodness-of-fit of the 2-PM model for the imipramine data.

**Conclusion:**

BQQ plots are sensitive to violations of the normality assumption for the random effects, suggesting that the approach is a useful tool for examining the goodness-of-fit of random-effects linear models when the goal is to measure individual treatment benefits.

## Background

Two-dimensional personalized medicine (2-PM) models are tools for measuring the severity of a patient’s chronic disease and the individual benefits of medical or behavioral treatments [1, 2]. The patient’s disease severity at a specific point in time is defined as the probability of missing the therapeutic target, and the individual’s benefit is therefore measured as the reduction in disease severity produced by the treatment. When the disease severity before treatment is close to 1, the patient is regarded as severely ill. The severity and individual benefits are functions of known and unknown patient’s characteristics. In practice, 2-PM models are built using linear regression models with random effects that are assumed to be normally distributed, and severities and benefits are calculated with both the fixed effects and the random effects of the model [1, 2]. In addition to being useful for measuring the individual benefits achieved by the patients of a clinical trial, the fitted 2-PM model can be used to measure individual benefits in potentially new patients [1, 2].

Given the potential applications of 2-PM models in personalized medicine, it is necessary to develop methods for examining their goodness-of-fit with a focus on their ability to measure individual benefits. In this article, we propose a graphical approach to assessing the goodness-of-fit of 2-PM models for continuous responses of severely ill patients. The approach compares the quantiles of the empirical Bayes (EB) predictors of individual treatment benefits of the patient sample against the theoretical quantiles of the distribution of individual benefits that are derived from the normality assumption for the random effects. We conducted a Monte Carlo simulation study that showed that the approach is sensitive to deviations from the normality assumption for the random effects. Specifically, the graphical approach captures the discrepancy between multivariate non-normal distributions for the random effects and normal distributions with the same mean and variance-covariance matrix. Since the main purpose of a 2-PM model is to measure the individual benefits of a medical or behavioral treatment in the patients of a clinical study or in potentially new patients [1, 2], the shape of our proposed goodness-of-fit plot will depend on the clinician’s therapeutic target. Therefore, conclusions on the degree of adequacy of the model will generally depend on the target.

Random-effects linear models (RELMs) are efficient tools for building 2-PM models of continuous responses. They are commonly used to understand individuals’ time trajectories of treatment effects [1–11]. In RELMs, the distributional assumption of the unobserved random effects is important for estimation and inference since the marginal likelihood function, obtained by integrating out the random effects, depends on the assumed distribution for the random effects. An EB approach is typically used to predict the random effects [1–3, 12–14]. In RELMs, the EB predictors of the random effects are estimates of the best linear unbiased predictors (BLUPs), which have optimality properties that do not require the normality assumption for the random effects [13]. Thus, a BLUP is robust to violations of the normality assumption and the EB predictor inherit some of its robustness [15–17]. The prediction accuracy of the BLUPs for random effects is not substantially affected by distribution misspecifications, as shown by both theoretical and numerical studies [16]. Traditionally and for ease of computation, in RELMs researchers assume that the unobserved random effects follow normal distributions. Violations of this normality assumption are possible. For instance, omission of patient level categorical covariates may produce multimodal distributions for the random intercept [18]. Although violations of the normality assumption have small-to-mild effects on the maximum likelihood estimates (MLE) of the fixed effects, they may affect the prediction of the random effects by increasing the bias of the variance components estimates, especially in generalized linear mixed effects models [12, 18–22]. Therefore, assessing the goodness-of-fit of 2-PM models with respect to the normality assumption for the random effects is crucial.

Several graphical methods have been proposed for examining the goodness-of-fit of random-effects models for longitudinal data. The most well known graphical approach is based on conditional residuals which are computed with EB predictors of the random effects [12, 23–26]. However, although conditional residuals may detect deviations from the assumption of linearity, are useful for detecting outliers and allow examining the normality assumption for the error term of the model, they do not allow examining the normality of the random effects. Normal quantile-quantile (QQ) plots are also commonly used in model development to examine the assumption of normality for the random effects. In normal QQ plots, the sample quantiles of the individual EB predictors of a random effect are plotted against the quantiles of a normal distribution whose mean and variance are the sample mean and variance of the EB predictors [24, 26, 27]. A limitation of normal QQ plots is that the data analyst must conduct a separate analysis for each random effect in the model. Moreover, there is some evidence that the EB predictors of a random effect tend to have a unimodal distribution, even in situations in which the true distribution of the random effect has two or more modes [15, 18, 19], which may artificially straighten the cloud of points of a normal QQ plot. Verbeke and Molenberghs [28] proposed a graphical diagnostic tool using gradient functions that checks the appropriateness of the random effects distribution assumption. Similar to normal QQ plots, a gradient function needs to be plotted for each random effect in the model. Pan and Lin [29] proposed graphical and numerical techniques based on cumulative sums of residuals for checking the link function and functional forms of covariates in generalized linear mixed models; their approach, however, does not address the assumption of normality for the random effects. Grady and Helms [30] assessed the fit of the assumed covariance structure by plotting lagged sample covariances or correlations. Diaz et al. [31] assessed the goodness-of-fit of a random intercept model by plotting random-effect-adjusted observations based on EB predictors of the random intercept versus expected observations. Although this approach is useful for examining the linearity assumption of RELMs and detecting outliers, it does not allow assessing the normality assumption for the random effects. Others have proposed formal statistical tests [32–35]. To check the normality assumption for the random effects, Efendi et al. [32] use a bootstrap test based on gradient functions. Drikvandi et al. [33] propose a diagnostic test based on Cramer-von Mises discrepancies. Alonso et al. [34] propose tests that use the eigenvalues of the variance-covariance matrices of fixed effects estimates obtained from robust inference methods. Similarly, Abad et al. [35] use information matrices to propose diagnostic tests for generalized linear mixed effects models.

This paper is organized as follows. First, we present a review of 2-PM models for continuous responses and the calculation of individual treatment benefits for severely ill patients. Then we present a motivation for the graphical approach and describe it in detail. Next, the approach is illustrated using data from a clinical trial of the antidepressant imipramine. Then we describe the simulation scenarios used to evaluate the performance of the proposed graphical approach. We also describe how Cramer-von Mises discrepancies are used to quantify deviations from the normality assumption for the random effects. The paper ends with a discussion and conclusions.

## Methods

### Individual severity and treatment benefits using time dependent 2-PM models

Time dependent 2-PM models allow understanding the evolution of individual treatment benefits over time [1]. Let *Y* be a continuous measure reflecting the patient’s disease. Before a treatment *Q* is initiated, the responses for patient *ω* are measured *k*_0,*ω*_ times and modeled by

$$ {Y}_{0,\omega, j}={\Lambda}_{\omega }+{\varepsilon}_{0,\omega, j},j=1,\dots, {k}_{0,\omega }. $$

After the treatment is initiated, the responses are measured *k*_1,*ω*_ times and modeled by

$$ {Y}_{Q,\omega, j}={\Lambda}_{\omega }+{\beta}_{Q,\omega, j}+{\varepsilon}_{\omega, j}^{\prime },j=1,\dots, {k}_{1,\omega }, $$

where Λ_*ω*_ = *α*_*ω*_ + ***λ***^*T*^***X***_*ω*_ and ${\beta}_{Q,\omega, j}={\theta}_{1,\omega }{t}_{\omega, j}+{\theta}_{2,\omega }{t}_{\omega, j}^2+\dots +{\theta}_{d,\omega }{t}_{\omega, j}^d$. Here, ***X***_*ω*_ is a vector of patient (subject) characteristics that do not change during the trial. For patient *ω*, Λ_*ω*_ is a constant number that reflects the patient’s disease state before treatment initiation and *β*_*Q*,*ω*,*j*_ is the individual’s (time-dependent) treatment effect after *t*_*ω*,*j*_ time units of treatment. We write *β*_*Q*,*ω*_(*t*) in place of *β*_*Q*,*ω*,*j*_ to express the treatment effect at a generic point in time *t*. We view *Λ*_*ω*_ and *β*_*Q*,*ω*_(*t*) as individual realizations of population-level random variables *Λ*^∗^ and ${\beta}_Q^{\ast }(t)$, respectively [1]. Also, *ε*_0,*ω*,*j*_ and ${\varepsilon}_{\omega, j}^{\prime }$ represent measurement errors or within-patient variability due to the patient’s internal or external factors, which we assume to be $N\left(0,{\sigma}_{\varepsilon}^2\right)$ and $N\left(0,{\sigma_{\varepsilon}^{\prime}}^2\right)$, respectively.

Here, we assume that the therapeutic target is to achieve *Y* ≤ *y*, where *y* is a value prespecified by the clinician. The patient’s basal disease severity is defined as the probability that the patient does not satisfy the therapeutic target before treatment initiation. Thus, a patient *ω* has basal severity [1, 2]

$$ {s}_{0,\omega }=1-\Phi \left(\frac{y-{\varLambda}_{\omega }}{\sigma_{\varepsilon }}\right), $$

where Φ is the cumulative distribution function of the standard normal distribution. The patient’s disease severity after a treatment duration *t* is

$$ {s}_{2,\omega }(t)=1-\Phi \left(\frac{y-{\varLambda}_{\omega }-{\beta}_{Q,\omega }(t)}{\sigma_{\varepsilon}^{\prime }}\right). $$

That is, *s*_2,*ω*_(*t*) is the probability that the target has not been achieved at time *t*. The patient’s individual benefit from the medical treatment at the point in time *t* is the reduction in disease severity

$$ {b}_{\omega }(t)={s}_{0,\omega }-{s}_{2,\omega }(t). $$

By definition, a patient is severely ill if the patients’ basal severity is approximately 1. Here, we assume that all patients are severely ill, that is, *s*_0,*ω*_ ≈ 1 for all *ω*. It is shown in Diaz [1] that under the reasonable assumption that ${\sigma}_{\varepsilon}^{\prime}\ge {\sigma}_{\varepsilon }$, if the patient is severely ill, the patient’s benefit can be computed as

1 $$ {b}_{\omega }(t)\approx \Phi \left(\frac{y-{\varLambda}_{\omega }-{\beta}_{Q,\omega }(t)}{\sigma_{\varepsilon}^{\prime }}\right). $$

In the following, we assume ${\sigma}_{\varepsilon}^{\prime }={\sigma}_{\varepsilon }$ which is usually clinically reasonable. Here, *α*_*ω*_ and *θ*_1,*ω*_, …, *θ*_*d*,*ω*_ are characteristic constants of patient *ω* that are viewed as realizations of random coefficients *α*^∗^ and ${\theta}_1^{\ast },\dots, {\theta}_d^{\ast }$ that do not necessarily have mean 0. In the terminology of mixed effects models, *E*(*α*^∗^), ***λ*** and $E\left({\theta}_1^{\ast}\right),\dots, E\left({\theta}_d^{\ast}\right)$ are the fixed effects, and *α*^∗^ − *E*(*α*^∗^) and ${\theta}_i^{\ast }-E\left({\theta}_i^{\ast}\right)$, *i* = 1, …, *d*, are the random effects which are usually assumed to be jointly normally distributed. Here, we propose a graphical method to examine the assumption of normality.

### Quantiles of individual benefits under the normality assumption

Under the assumption of normality for the random effects, since the patients are severely ill, the cumulative distribution function of individual benefits for patients with covariate value ***X*** = ***x*** at time *t* is [1]

2 $$ F(z)=F\left(z;\boldsymbol{x},t\right)=\Phi \left(\frac{\Phi^{-1}(z)-\mu }{\gamma}\right),0<z<1, $$

where $\mu =\mu \left(\boldsymbol{x},t\right)=\frac{y-E\left({\varLambda}^{\ast }+{\beta}_Q^{\ast }(t)\right)}{\sigma_{\varepsilon}^{\prime }}$ and ${\gamma}^2={\gamma}^2(t)=\frac{\mathrm{Var}\left({\varLambda}^{\ast }+{\beta}_Q^{\ast }(t)\right)}{{\sigma_{\varepsilon}^{\prime}}^2}$. Further, the *p*-th quantile of the probability distribution function of individual treatment benefits is [1]

3 $$ B(p)=B\left(p;\boldsymbol{x},t\right)=\Phi \left(\gamma {\Phi}^{-1}(p)+\mu \right),0<p<1. $$

The quantities in (2) and (3) are functions of treatment duration *t*, since *μ* and *γ* are. They also vary with the fixed effects and variance components (i.e., the variances and covariances of the random effects and the error variance).

### A motivation for the proposed graphical approach

Here, we estimate (predict) the individual treatment benefits using the EB approach described in Diaz [1, 2]. The EB predictors of individual treatment benefits are obtained by replacing the fixed effects, error variance and individual random effects in Eq. (1) with their estimates or predictors. The fixed effects and variance components are usually estimated through maximum or restricted maximum likelihood [4–6, 12, 13, 36]. We predict the random effects following an EB approach [1, 2, 9, 12, 13, 19, 36–38]. Importantly, the EB predictors of random effects are estimates of the best linear unbiased predictors (BLUPs) and the BLUPs do not assume normality for the random effects [13]. Moreover, the EB predictors of random effects are relatively robust to violations of the normality assumption [13, 15, 16, 38]. Because of this, one can view the sample quantiles of the EB-predicted individual benefits as robust estimates of the quantiles of the probability distribution of individual benefits. Alternatively, we can directly estimate the quantiles by replacing the fixed effects and variance components in Eq. (3) with their corresponding estimates. Therefore, if the normality assumption is violated, we expect the quantiles estimated with Eq. (3) to be substantially different from the sample quantiles based on the BLUPs because Eq. (3) is derived from the assumption of normality. Thus, we propose to compare the sample quantiles based on the BLUPs with the quantiles calculated with Eq. (3) in order to evaluate the assumption of normality for the random effects.

### Goodness-of-fit plot

Suppose the sample of patients can be divided into *G* subgroups. This is possible, for instance, when the patient characteristics are categorical or when a characteristic is continuous and it is split into categories based on published cut-off values or percentiles. Therefore, we assume that ***X***_*ω*_ includes only binary (dummy) covariates and that ***X***_*ω*_ has *G* distinct possible values ***x***_1_, …, ***x***_*G*_. Let *N*_*g*_ be the number of patients in the subpopulation of patients for whom ***X***_*ω*_ = ***x***_*g*_, and let $N=\sum \limits_{g=1}^G{N}_g$ be the total number of patients. For a particular time *t*, let ${\hat{b}}_{g,t,1},\dots, {\hat{b}}_{g,t,{N}_g}$ be the EB-predicted individual benefits for the *N*_*g*_ patients in group *g*, and ${\hat{b}}_{g,t,(1)}<{\hat{b}}_{g,t,(2)}<\dots <{\hat{b}}_{g,t,\left({N}_g\right)}$ be the corresponding order statistics. A benefit quantile-quantile (BQQ) plot consists of plotting in an x-y plane the *N* points

$$ \left(\hat{B}\left(\frac{i-0.5}{N_g};{\boldsymbol{x}}_g,t\right),{\hat{b}}_{g,t,(i)}\right),i=1,\dots, {N}_g,g=1,\dots, G. $$

where $\hat{B}$ is obtained by replacing fixed effects and variance components in Eq. (3) with their maximum likelihood or restricted maximum likelihood estimates (RMLEs). Thus, a BQQ plot compares the sample quantiles of individual benefits predicted with the EB approach versus estimates of the theoretical quantiles derived from the normality assumption for the random effects. In practice, we use the maximum point in time available in the dataset as a value for *t*.

If the points on the BQQ plot do not deviate asymmetrically much about the *y* = *x* line, then we conclude that the normality assumption for the random effects of the 2-PM model is appropriate and, therefore, that we can have reasonable confidence in the EB predictors of the individual benefits achieved by the patient sample. Note that, by Eqs. (1) and (3), the shape of the BQQ plot depends on the prespecified therapeutic target and, therefore, conclusions on the adequacy of the 2-PM model are applicable only to the specific target used.

### Application to depression study

As an illustration, we use clinical trial data from 66 patients under imipramine treatment with two types of depression diagnosis [39]. The diagnoses were endogenous (*N*_1_ = 37) or nonendogenous (*N*_2_ = 29). The data is available in Hedeker and Gibbons [6] and is also analyzed by Diaz [1]. The response variable, the Hamilton Rating Scale (HRS) for depression, was recorded at the beginning and end of the week before imipramine treatment initiation and at the end of each of the next 4 weeks during treatment. Diaz [1] fitted a random-effects linear model of the HRS scores in order to predict individual treatment benefits but did not provide evidence for the model’s goodness-of-fit which we examine here. As covariates, the model included diagnosis (1 = endogenous, 0 = nonendogenous) as well as time and time-square, where time is the number of weeks on treatment. Gender was not significant after adjusting for diagnosis and was therefore not included as a covariate. The intercept and the linear and quadratic terms of time had random effects in addition to the fixed effects. We assumed an unstructured covariance matrix for the random effects and homoscedastic independent errors. The SAS procedure MIXED, which assumes normally distributed random effects, was used to obtain the MLEs of the fixed effects and EB predictors for the random effects for all patients (SAS Institute Inc. Cary, NC). All observed baseline HRS scores were ≥11. For the computation of individual imipramine benefits, we assumed that the therapeutic target was to achieve an HRS score ≤7 (i.e., *y* = 7). Using this target and the model parameter estimates, Diaz [1] calculated that the EB predictors of all patients’ basal severities were approximately 1 and, therefore, concluded that the patients can be assumed to be severely ill.

### Results for the application study

Parameter estimates are shown in Table 1 in Diaz [1]. Figure S1 in the Supplementary Information indicates that for the depression data, the model satisfies reasonably well the normality assumption for the residuals.

**Table 1:** Summary of simulation scenarios for evaluating the performance of BQQ plots

	Scenario 1	Scenario 2	Scenario 3	Scenario 4
Model:	1 (Equation 4)	1 (Equation 4)	2 (Equation 5)	2 (Equation 5)
Number of patients (*N*)	{30, 50, 100, 150, 200, 300, 500}	{20, 60, 100, 160, 200, 300, 500}	{30, 50, 100, 150, 200, 300, 500}	{30, 50, 100, 150, 200, 300, 500}
# of measurements per patient, *n* = *k*_0,*ω*_ + *k*_1,*ω*_	For all scenarios, when *k*_1,*ω*_ = 2, *n* = 4*;* when *k*_1,*ω*_ = 4*, n* = 6.			
Binary covariate	*x*_*ω*_~Bernoulli(0.6) (for all scenarios)			
Measurement errors	$\mathrm{i}.\mathrm{i}.\mathrm{d}.,{\varepsilon}_{\omega, j}\sim N\left(0,{\sigma}_{\varepsilon}^2=10\right)$ (for all scenarios)			
Fixed effects (***ψ***)	(21, 2, −5, 0.5)^*T*^	(21, 2, −5, 0.5)^*T*^	(21.4, 1.92, −3.97, 0.35)^*T*^	(24, 1.92, 0.97, −0.35)^*T*^
Non-normal random effects	${\boldsymbol{\tau}}_{\omega}\sim \frac{1}{2}N\left({\boldsymbol{m}}_1^{\ast }=w\bullet {\boldsymbol{m}}_1,V\right)$ $+\frac{1}{2}N\left({\boldsymbol{m}}_2^{\ast }=w\bullet {\boldsymbol{m}}_2,V\right)$ ***m***_1_ = (0, −1)^*T*^, ***m***_2_ = (0, 1)^*T*^ $$ V=\left[\begin{array}{cc}1& 0.9\\ {}0.9& 1\end{array}\right] $$ *w* ∈ {1, 2, 3, 4, 5}	${\boldsymbol{\tau}}_{\omega}\sim \frac{3}{4}N\left({\boldsymbol{m}}_1,V\right)+\frac{1}{4}N\left({\boldsymbol{m}}_2,V\right)$ ***m***_1_ = (0, −1)^*T*^, ***m***_2_ = (0, 3)^*T*^ $$ V=\left[\begin{array}{cc}{\sigma}_1^2& 0.9\\ {}0.9& {\sigma}_2^2\end{array}\right] $$ ${\sigma}_1^2={\sigma}_2^2\in \left\{1,2,3,4,5\right\}$	***τ***_*ω*_~*t*_*v*_(***m***, Γ) *v* ∈ {3, 5, 7, 9, 11, 13} ***m*** = (0, 0, 0)^*T*^ $$ \Gamma =\left[\begin{array}{ccc}10.4& 0.279& -0.341\\ {}0.279& 13.06& -2.466\\ {}-0.341& -2.466& 0.581\end{array}\right] $$	${\boldsymbol{\tau}}_{\omega}\sim \frac{1}{2}N\left({\boldsymbol{m}}_1^{\ast }=w\bullet {\boldsymbol{m}}_1,V\right)$ $+\frac{1}{2}N\left({\boldsymbol{m}}_2^{\ast }=w\bullet {\boldsymbol{m}}_2,V\right)$ ***m***_1_ = (0, −1, 1)^*T*^ ***m***_2_ = (0, 1, −1)^*T*^ $$ V=\left[\begin{array}{ccc}10.4& 0.279& -0.341\\ {}0.279& 13.06& -2.466\\ {}-0.341& -2.466& 0.581\end{array}\right] $$*w* ∈ {0.5, 1, 1.5, 2, 2.5, 3}
Reference normal random effects^a^	***τ***_*ω*_~*N*(***m***, *D*^∗^) ***m*** = (0, 0)^*T*^ ${D}^{\ast }=\frac{1}{2}{\boldsymbol{m}}_1^{\ast }{{\boldsymbol{m}}_1^{\ast}}^T+\frac{1}{2}{\boldsymbol{m}}_2^{\ast }{{\boldsymbol{m}}_2^{\ast}}^T+V$	***τ***_*ω*_~*N*(***m***, *D*^∗^) ***m*** = (0, 0)^*T*^ ${D}^{\ast }=\frac{3}{4}{\boldsymbol{m}}_1{\boldsymbol{m}}_1^T+\frac{1}{4}{\boldsymbol{m}}_2{\boldsymbol{m}}_2^T+V$	***τ***_*ω*_~*N*(***m***, *D*^∗^) ***m*** = (0, 0, 0)^*T*^ ${D}^{\ast }=\left(\frac{v}{v-2}\right)\Gamma$	***τ***_*ω*_~*N*(***m***, *D*^∗^) ***m*** = (0, 0, 0)^*T*^ ${D}^{\ast }=\frac{1}{2}{\boldsymbol{m}}_1^{\ast }{{\boldsymbol{m}}_1^{\ast}}^T+\frac{1}{2}{\boldsymbol{m}}_2^{\ast }{{\boldsymbol{m}}_2^{\ast}}^T+V$

^a^The reference normal distribution is a distribution with the same mean and variance-covariance matrix as the corresponding non-normal distribution. BQQ plots and CVM discrepancies computed with a non-normal distribution were compared with those of its reference distribution

Figure S2 in the Supplementary Information shows histograms and kernel densities of EB predictors of the random effects. The shapes of the histograms seem to suggest approximate normality for the distribution of the random effects. However, Verbeke and Lesaffre [19] and Mcculloch and Neuhaus [15] have found that the shape of the histograms for EB predictors may be misleading and may not reflect the true distribution of the random effects.

**Fig. 1:**
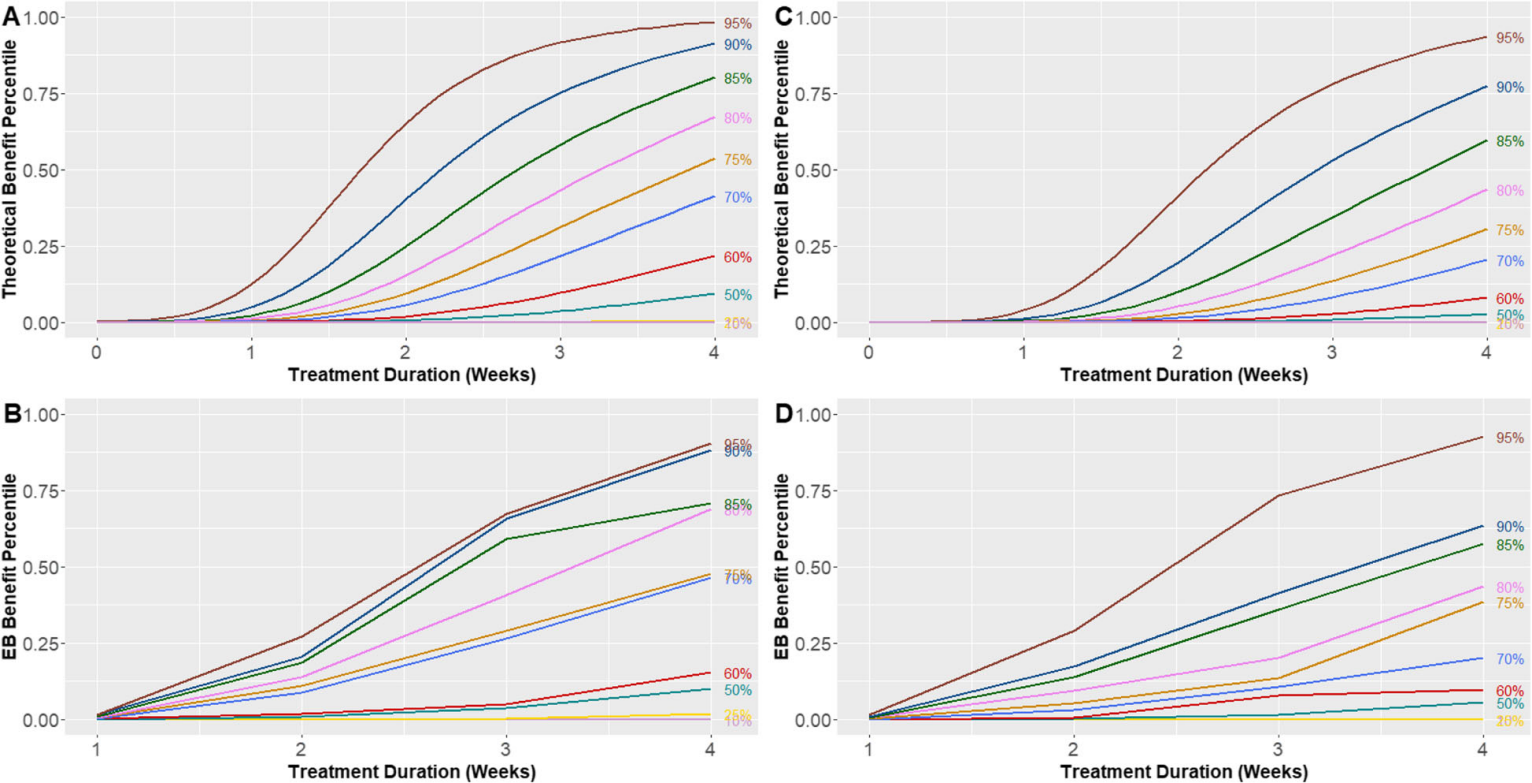
Selected *p* × 100% percentiles of the probability distribution of individual benefits of imipramine treatment as functions of treatment duration in patients with nonendogenous diagnosis (left panels; **a**, **b**) and endogenous diagnosis (right panels; **c**, **d**), estimated with two different methods, for *p*= 0.1, 0.25, 0.5, 0.6, 0.7, 0.75, 0.8, 0.85, 0.90, 0.95. Upper panels (**a**, **c**): percentiles estimated with Eq. (3) which assumes normality for the random effects. Lower panels (**b**, **d**): sample percentiles of EB predictors of individual benefits

We used Eq. (3) to estimate selected *p* × 100% percentiles of the individual benefits of imipramine as functions of treatment duration for nonendogenous (Fig. 1a) and endogenous (Fig. 1c) patients. For comparison purposes, we also calculated the corresponding sample percentiles of the EB-predicted individual benefits for nonendogenous (Fig. 1b) and endogenous (Fig. 1d) patients. In general, the sample percentiles and the estimated theoretical percentiles seem to convey similar information. For instance, for fixed values of *p*, both estimation methods show that the benefit percentiles for nonendogenous patients tended to be higher than the corresponding percentiles for endogenous patients, which reflects the fact that imipramine was more beneficial in nonendogenous than endogenous patients [1].

**Fig. 2:**
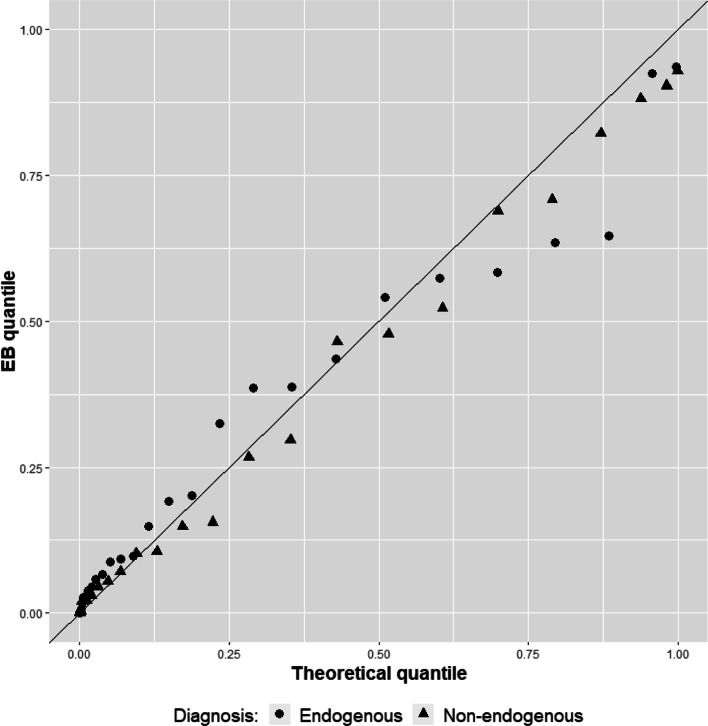
BQQ plot of individual treatment benefits after 4 weeks of imipramine treatment for patients with depression

The BQQ plot in Fig. 2, however, displays a better comparison of the estimated theoretical percentiles and sample percentiles at week 4. The points in the plot are distributed closely about the *y* = *x* line, without exhibiting any considerable asymmetric deviations. Figure 2 also exhibits a relatively high concentration of patients at the bottom left corner. The EB quantiles coincided with the theoretical quantiles in that about 59% of the patients achieved relatively small individual imipramine benefits ≤0.13 in a probability scale. Thus, the sample quantiles of the patients’ individual benefits matched closely the theoretical quantiles derived from the normality assumption. This suggests the adequacy of this assumption and the goodness-of-fit of the 2-PM model for the imipramine data, provided the purpose of the model is to measure individual benefits under the therapeutic target HRS ≤7.

**Fig. 3:**
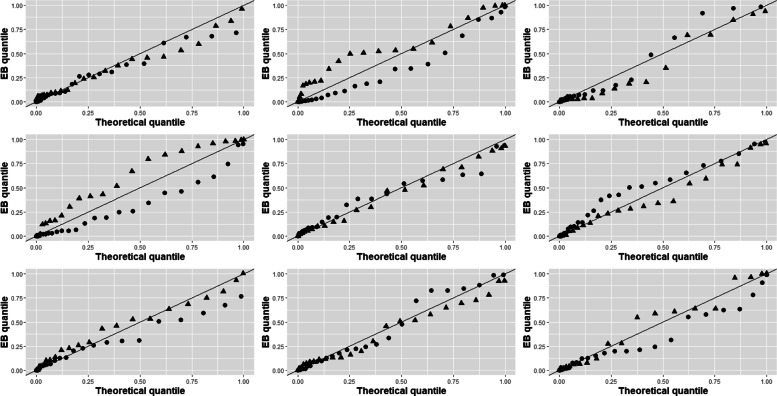
Comparison of the BQQ plot for the imipramine data versus eight BQQ plots that were consecutively simulated with the fitted imipramine model. The BQQ plot for the real data is in the middle and the other plots were computed with simulated patients using normal random effects. Circles (●) and triangles (▲) correspond to endogenous and non-endogenous patients, respectively

Figure 3 compares the BQQ plot in Fig. 2 with eight BQQ plots that were consecutively simulated with the fitted model of HRS scores, which had normal random effects. The plot in the middle of Fig. 3 is the BQQ plot computed with the real data and the other plots are the simulated ones. The parameters in Table 1 of [1] were used to simulate the patients of the simulated BQQ plots and, to ensure comparability with the real data, 37 simulated endogenous and 29 simulated non-endogenous patients were used in each simulated plot. Note that the plot made with the real data cannot be singled out easily as most different from the other plots and does not seem to have a distinguishing feature compared to the other plots. This reinforces our conclusion that the assumption of normality for the random effects of the 2-PM model is reasonable [40, 41].

We also applied the diagnostic tests proposed by Alonso et al. [34] to this dataset. The null hypothesis is that the normality assumption for the random effects is reasonable. The two determinant tests and the determinant-trace test yielded the test statistics *δ*_*d*1_ = 2.9, *δ*_*d*2_ = 1.19 and *δ*_*d*3_ = 1.28, with corresponding *p*-values of 0.085, 0.276 and 0.258. All three *p*-values were larger than the chosen 0.05 significant level. This is consistent with the conclusions from our proposed graphical approach. We include here these tests only for comparison purposes and do not consider them as a final confirmatory tool (see the Discussion Section).

### Simulation study

We conducted a simulation study to assess the performance of BQQ plots under violations of the normality assumption for the random effects. Motivated by the application study in Diaz [1], data from the following two models were simulated:

#### **Model 1:** (Random intercept and random slope for time)

4 $$ {\displaystyle \begin{array}{c}{Y}_{\omega, j}^{\prime }={\psi}_0+{\psi}_1{x}_{\omega }+{\psi}_2{t}_{\omega, j}+{\psi}_3{t}_{\omega, j}^2+{\tau}_{0,\omega }+{\tau}_{2,\omega }{t}_{\omega, j}+{\varepsilon}_{\omega, j},\\ {}\kern0ex \omega =1,\dots, N,j=1,\dots, n,\end{array}} $$

such that *Λ*_*ω*_ = *ψ*_0_ + *τ*_0,*ω*_ + *ψ*_1_*x*_*ω*_ and *β*_*Q*,*ω*_(*t*) = (*ψ*_2_ + *τ*_2,*ω*_)*t* + *ψ*_3_*t*^2^, *N* is the number of patients and *n* is the number of observations per patient. Here, ***ψ*** = (*ψ*_0_, *ψ*_1_, *ψ*_2_, *ψ*_3_)^*T*^ are the fixed effects and ***τ***_*ω*_**=** (*τ*_0,*ω*_, *τ*_2,*ω*_)^*T*^ are the random effects with mean 0. Moreover, *x*_*ω*_~Bernoulli(0.6) represents a patient’s time-independent characteristic (for instance, gender, smoking, etc.) and the *ε*_*ω*,*j*_ ’s are mutually independent with ${\varepsilon}_{\omega, j}\sim N\left(0,{\sigma}_{\varepsilon}^2=10\right)$.

#### **Model 2:** (Random intercept and random slopes for time and time square)

5 $$ {Y}_{\omega, j}^{\prime }={\psi}_0+{\psi}_1{x}_{\omega }+{\psi}_2{t}_{\omega, j}+{\psi}_3{t}_{\omega, j}^2+{\tau}_{0,\omega }+{\tau}_{2,\omega }{t}_{\omega, j}+{\tau}_{3,\omega }{t}_{\omega, j}^2+{\varepsilon}_{\omega, j}, $$

$$ \omega =1,\dots, N,j=1,\dots, n, $$

such that *Λ*_*ω*_ = *ψ*_0_ + *τ*_0,*ω*_ + *ψ*_1_*x*_*ω*_ and *β*_*Q*,*ω*_(*t*) = (*ψ*_2_ + *τ*_2,*ω*_)*t* + (*ψ*_3_ + *τ*_3,*ω*_)*t*^2^. In this case, ***τ***_*ω*_**=** (*τ*_0,*ω*_, *τ*_2,*ω*_, *τ*_3,*ω*_)^*T*^ are the random effects with mean 0. We assumed an unstructured variance-covariance matrix for the random effects for both models [5, 6] and no missing responses.

Varying values for *N* were used and *n* = 4 or 6. For either model, we simulated 2 baseline measurements and 2 or 4 measurements under medical treatment. Thus, for all models, *k*_0,*ω*_ = 2, and *t*_*ω*,1_ = *t*_*ω*,2_ = 0. When *n* = 4, *k*_1,*ω*_ = 2, *t*_*ω*,3_ = 1 and *t*_*ω*,4_ = 4; and when *n* = 6, *k*_1,*ω*_ = 4, *t*_*ω*,3_ = 1, *t*_*ω*,4_ = 2, *t*_*ω*,5_ = 3 and *t*_*ω*,6_ = 4. For all models, ${Y}_{0,\omega, j}={Y}_{\omega, j}^{\prime }$ for *j* = 1, 2, and ${Y}_{Q,\omega, j}={Y}_{\omega, j+2}^{\prime }$ for *j* = 1, …, *k*_1,*ω*_.

The therapeutic target was to achieve *Y* ≤ *y* with *y* = 7. The MLEs of ***ψ*** and ${\sigma}_{\varepsilon}^2$ are denoted by $\hat{\boldsymbol{\psi}}={\left({\hat{\psi}}_0,{\hat{\psi}}_1,{\hat{\psi}}_2,{\hat{\psi}}_3\right)}^T$ and ${\hat{\sigma}}_{\varepsilon}^2$; and the EB predictor of ***τ***_*ω*_ by ${\hat{\boldsymbol{\tau}}}_{\omega }={\left({\hat{\tau}}_{0,\omega },{\hat{\tau}}_{2,\omega}\right)}^T$ for Model 1 or ${\hat{\boldsymbol{\tau}}}_{\omega }={\left({\hat{\tau}}_{0,\omega },{\hat{\tau}}_{2,\omega },{\hat{\tau}}_{3,\omega}\right)}^T$ for Model 2. Here, we investigate BQQ plots computed at the last time point, namely *t* = 4. We used Eq. (1) to predict the individual benefits after replacing *σ*_*ε*_, Λ_*ω*_ and *β*_*Q*,*ω*_(*t*) with their estimates ${\hat{\sigma}}_{\varepsilon }$, ${\hat{\Lambda}}_{\omega }={\hat{\psi}}_0+{\hat{\tau}}_{0,\omega }+{\hat{\psi}}_1{x}_{\omega }$, and ${\hat{\beta}}_{Q,\omega }(t)=\left({\hat{\psi}}_2+{\hat{\tau}}_{2,\omega}\right)t+{\hat{\psi}}_3{t}^2$ for Model 1 or ${\hat{\beta}}_{Q,\omega }(t)=\left({\hat{\psi}}_2+{\hat{\tau}}_{2,\omega}\right)t+\left({\hat{\psi}}_3+{\hat{\tau}}_{3,\omega}\right){t}^2$ for Model 2.

Let Σ_*i*,*j*_ be the (*i*, *j*)-th entry of the variance-covariance matrix of ***τ***_*ω*_ and ${\hat{\Sigma}}_{i,j}$ be its maximum likelihood estimator. Thus, Σ_*i*,*j*_ is of dimension 2 × 2 for Model 1, and 3 × 3 for Model 2. The *μ* in Eq. (3) is estimated with $\hat{\mu}=\left(y-\left({\hat{\psi}}_0+{\hat{\psi}}_1{x}_{\omega }+{\hat{\psi}}_2t+{\hat{\psi}}_3{t}^2\right)\right)/{\hat{\sigma}}_{\varepsilon }$ for both models, whereas *γ* is estimated with ${\hat{\gamma}}^2=\left({\hat{\Sigma}}_{1,1}+{t}^2{\hat{\Sigma}}_{2,2}+2t{\hat{\Sigma}}_{1,2}\right)/{\hat{\sigma}}_{\varepsilon}^2$ for Model 1, and ${\hat{\gamma}}^2=\left({\hat{\Sigma}}_{1,1}+{t}^2{\hat{\Sigma}}_{2,2}+{t}^4{\hat{\Sigma}}_{3,3}+2t{\hat{\Sigma}}_{1,2}+2{t}^2{\hat{\Sigma}}_{1,3}+2{t}^3{\hat{\Sigma}}_{2,3}\right)/{\hat{\sigma}}_{\varepsilon}^2$ for Model 2.

Table 1 shows the “true” fixed effects employed in simulations. These were chosen so that most simulated patients were severely ill under all examined non-normal and normal distributions for the random effects; specifically, *P*(*s*_0,*ω*_ > 0.9) ≥ 0.95.

### Simulation of random effects

We implemented four simulation scenarios to represent situations in which the normality assumption for the random effects is violated (Table 1). For comparison purposes, in each scenario, ***τ***_*ω*_ was simulated from both a non-normal distribution and a reference normal distribution with the same mean and variance-covariance matrix.

#### Scenario 1: Model 1 with a symmetric mixture of two bivariate normal distributions

**Fig. 4:**
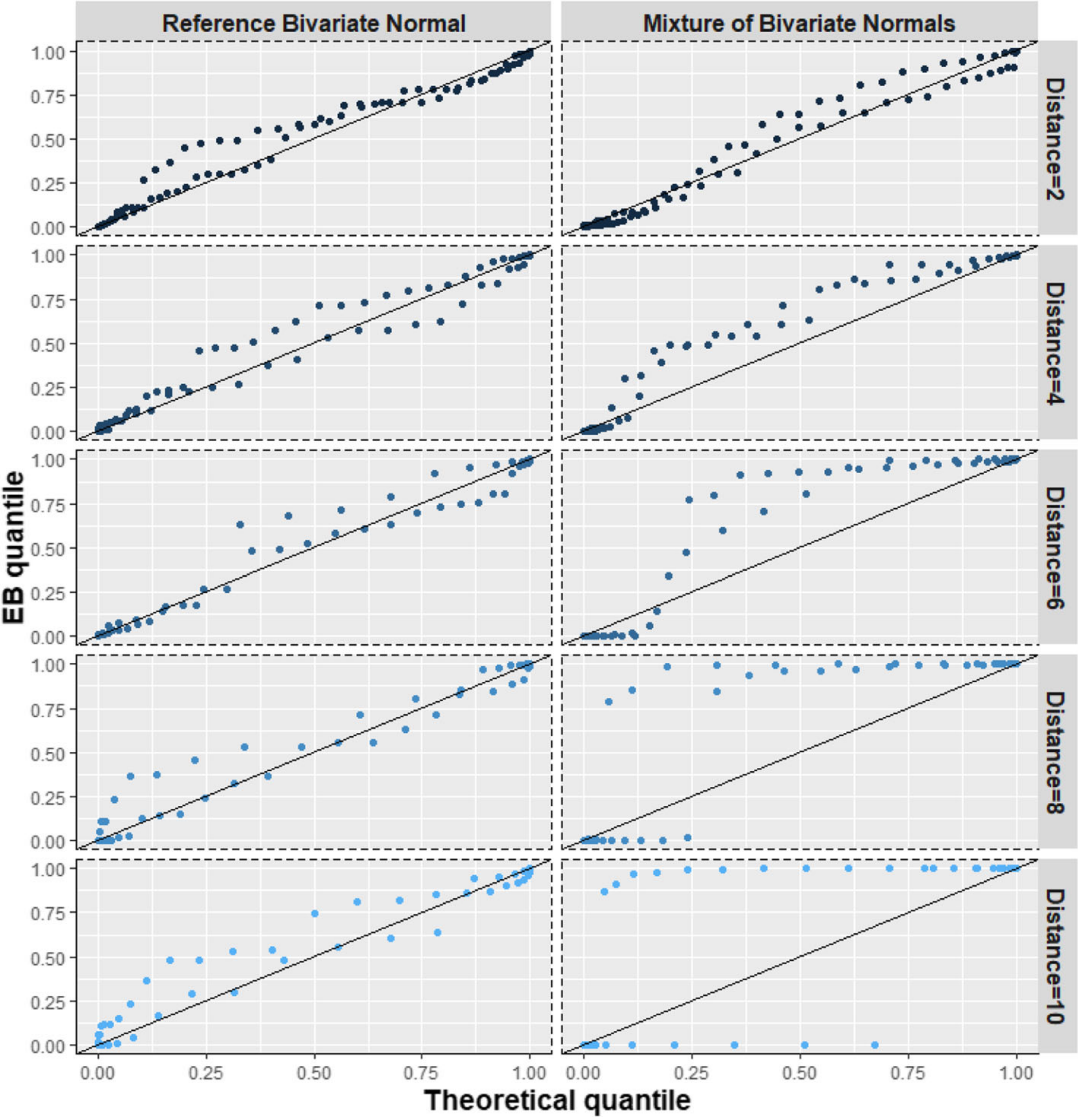
(Scenario 1). Benefit quantile-quantile (BQQ) plots of simulated treatment benefits at *t* = 4 for *N* = 100 patients with *n* = 6 measures per patient. The plots on the right panel correspond to random effects simulated from symmetric mixtures of two bivariate normal distributions whose mean vectors were separated by distances of 2, 4, 6, 8 or 10. The left panels correspond to random effects simulated from bivariate normal distributions with the same mean and variance-covariance matrix as the corresponding non-normal distribution on the same row at the right panel

Here, we explore the effect on the BQQ plot of the distance between the means of the two components of a mixture of normal distributions for *N* ∈ {30, 50, 100, 150, 200, 300, 500}. The true ***τ***_*ω*_ was distributed as

$$ {\boldsymbol{\tau}}_{\omega}\sim \frac{1}{2}N\left({\boldsymbol{m}}_1^{\ast }=w\bullet {\boldsymbol{m}}_1,V\right)+\frac{1}{2}N\left({\boldsymbol{m}}_2^{\ast }=w\bullet {\boldsymbol{m}}_2,V\right) $$

where ***m***_1_ = (0, −1)^T^, ***m***_2_ = (0, 1)^T^ and $$ V=\left[\begin{array}{cc}1& 0.9\\ {}0.9& 1\end{array}\right] $$. The distance between the mean vectors is $w\bullet \sqrt{{\left({\boldsymbol{m}}_1-{\boldsymbol{m}}_2\right)}^T\left({\boldsymbol{m}}_1-{\boldsymbol{m}}_2\right)}.$ We examined *w* ∈ {1, 2, 3, 4, 5}. The reference normal distribution with the same mean and variance-covariance matrix was *N*(***m***, *D*^∗^), where ***m*** = (0, 0)^T^ and ${D}^{\ast }=\frac{1}{2}{\boldsymbol{m}}_1^{\ast }{{\boldsymbol{m}}_1^{\ast}}^T+\frac{1}{2}{\boldsymbol{m}}_2^{\ast }{{\boldsymbol{m}}_2^{\ast}}^T+V$. Here, a greater distance between the means of the two component distributions represents a greater deviation from normality and, therefore, we expect the BQQ plot to show greater departures from the diagonal line (Figs. 4, 8; Table S1).

#### Scenario 2: Model 1 with an asymmetric mixture of two bivariate normal distributions for the random effects

**Fig. 5:**
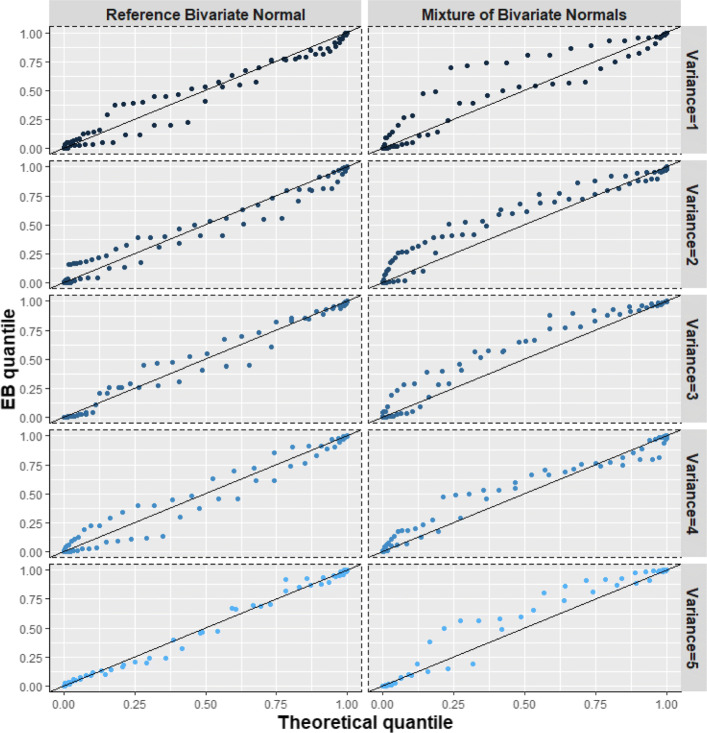
(Scenario 2). Benefit quantile-quantile (BQQ) plots of simulated treatment benefits at *t* = 4 for *N* = 100 patients with *n* = 6 measures per patient. The plots on the right panel correspond to random effects simulated from asymmetric mixtures of two bivariate normal distributions. Either bivariate component had variances ${\sigma}_1^2={\sigma}_2^2$ with values 1, 2, 3, 4 or 5. The left panels correspond to random effects simulated from bivariate normal distributions with the same mean and variance-covariance matrix as the corresponding non-normal distribution on the same row at the right panel

Here, we explore how the variance of the components of a mixture of normal distributions affects the BQQ plot, for sample sizes *N* ∈ {20, 60, 100, 160, 200, 300, 500}. The true random effects vector ***τ***_*ω*_ was distributed as

$$ {\boldsymbol{\tau}}_{\omega}\sim \frac{3}{4}N\left({\boldsymbol{m}}_1,V\right)+\frac{1}{4}N\left({\boldsymbol{m}}_2,V\right) $$

where ***m***_1_ = (0, −1)^T^, ***m***_2_ = (0, 3)^T^ and $$ V=\left[\begin{array}{cc}{\sigma}_1^2& 0.9\\ {}0.9& {\sigma}_2^2\end{array}\right] $$. We examined ${\sigma}_1^2={\sigma}_2^2\in \left\{1,2,3,4,5\right\}$. In this case, the overall mean and variance are ***m*** = (0, 0)^T^ and ${D}^{\ast }=\frac{3}{4}{\boldsymbol{m}}_1{\boldsymbol{m}}_1^T+\frac{1}{4}{\boldsymbol{m}}_2{\boldsymbol{m}}_2^T+V$. Thus, for comparison purposes, ***τ***_*ω*_ was also simulated from the reference bivariate *N*(***m***, *D*^∗^). Here, since the mean vectors are fixed, a greater variance for the components of the mixture implies a “less bimodal” distribution. Therefore, we expect BQQ plots for the non-normal cases to be more like their corresponding reference normal cases when the variability of the components is larger (Figs. 5, 9; Table S2).

#### Scenario 3: Model 2 with a trivariate t distribution for the random effects

**Fig. 6:**
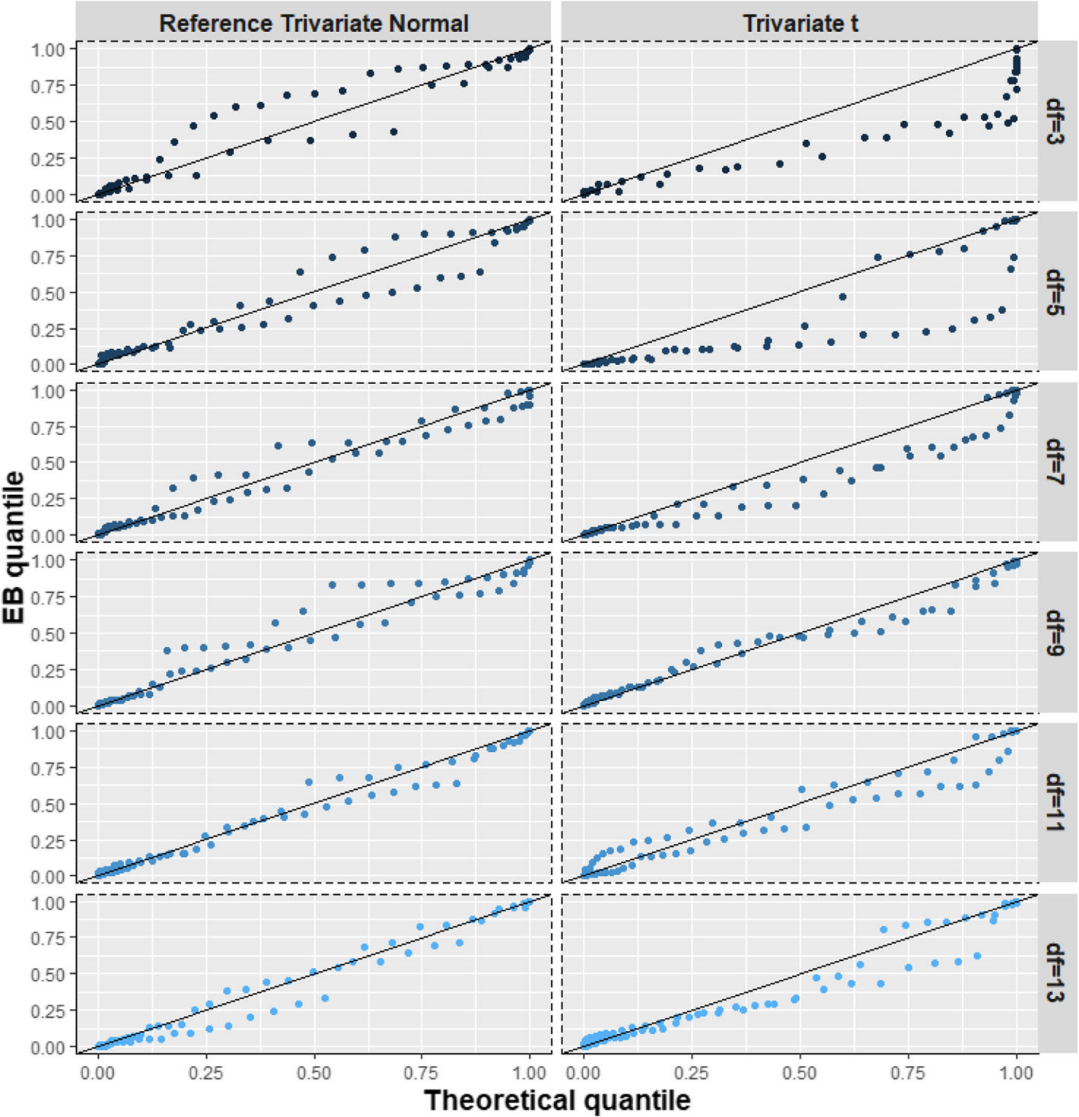
(Scenario 3). Benefit quantile-quantile (BQQ) plots of simulated treatment benefits at *t* = 4 for *N* = 100 patients with *n* = 6 measures per patient. The plots on the right panel correspond to random effects simulated from trivariate t distributions with degrees of freedom (df) of 3, 5, 7, 9, 11 or 13. The left panels correspond to random effects simulated from trivariate normal distributions with the same mean and variance-covariance matrix as the corresponding non-normal distribution on the same row at the right panel

Here, the true random effects were simulated from a trivariate t distribution with degrees of freedom *v* ∈ {3, 5, 7, 9, 11, 13}, location parameter ***m*** = (0, 0, 0)^T^, and shape parameter Γ given in Table 1. The purpose was to study how BQQ plots are affected by the heaviness of the tails of the t distribution, using *N* ∈ {30, 50, 100, 150, 200, 300, 500}. The reference normal distribution with the same mean and variance-covariance matrix was $N\left(\boldsymbol{m},{D}^{\ast }=\left(\frac{v}{v-2}\right)\Gamma \right)$ [42]. In this scenario, smaller degrees of freedom are associated with heavier tails for the distribution of random effects. Thus, we expect BQQ plots for non-normal cases to resemble more the reference normal plots when *v* is larger (Figs. 6, 10; Table S3).

#### Scenario 4: Model 2 with a symmetric mixture of two trivariate normal distributions

**Fig. 7:**
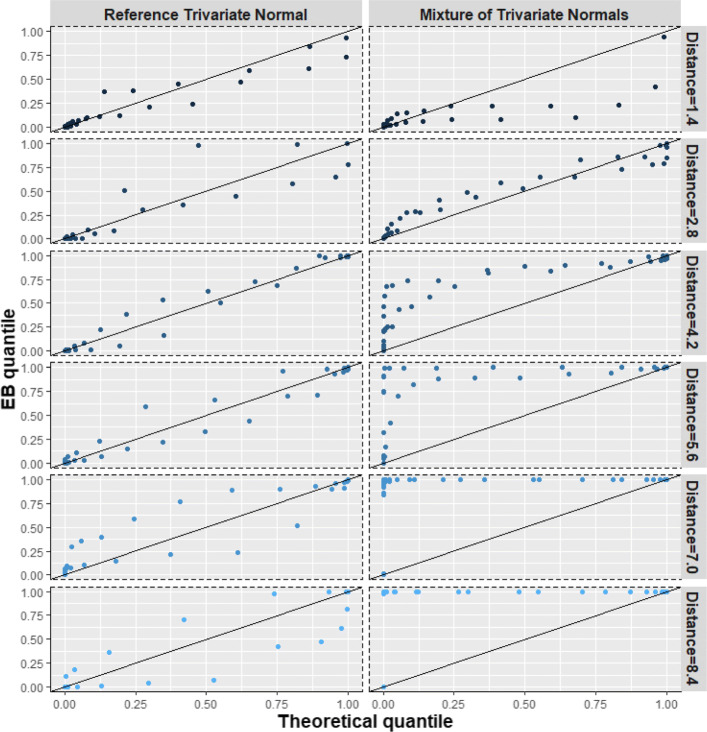
(Scenario 4). Benefit quantile-quantile (BQQ) plots of simulated treatment benefits at *t* = 4 for *N* = 100 patients with *n* = 6 measures per patient. The plots on the right panel correspond to random effects simulated from mixtures of two trivariate normal distributions whose mean vectors were separated by distances of 1.4, 2.8, 4.2, 5.6, 7.0 or 8.4. The left panels correspond to random effects simulated from trivariate normal distributions with the same mean and variance-covariance matrix as the corresponding non-normal distribution on the same row at the right panel

This scenario is analogous to Scenario 1, except that we used trivariate normal distributions for the components of the mixture. The goal was also to examine the effect of the distance between the means of the two normal components on BQQ plots. Since a greater distance represents a greater deviation from normality, we expect the BQQ plots to show greater departures from the diagonal line (Figs. 7, 11; Table S4).

### Cramer-von Mises discrepancy measure

We used Cramer-von Mises discrepancy measure (CVM) to quantify the deviation of the BQQ plot from the y = x line under violations of the normality assumption [43–45]. Let ${F}_{N_g}(z)={F}_{N_g}\left(z;t\right)$ be the empirical distribution function of ${\hat{b}}_{g,t,1},\dots, {\hat{b}}_{g,t,{N}_g}$ and denote ${U}_{g,t,1}=F\left({\hat{b}}_{g,t,1};{\boldsymbol{x}}_g,t\right),\dots, {U}_{g,t,{N}_g}=F\left({\hat{b}}_{g,t,{N}_g};{\boldsymbol{x}}_g,t\right)$. The CVM discrepancy between ${F}_{N_g}\left(z;t\right)$ and *F*(*z*; ***x***_*g*_, *t*) was computed as [44].

$$ {\Omega}_{g,t}={\int}_{-\infty}^{+\infty }{\left\{{F_N}_g\left(z;t\right)-F\left(z;{\boldsymbol{x}}_g,t\right)\right\}}^2 dF\left(\mathrm{z};{\boldsymbol{x}}_g,t\right)=\frac{1}{12{N}_g^2}+\frac{1}{N_g}\sum \limits_{k=1}^{N_g}{\left({U}_{g,t,k}-\frac{2k-1}{2{N}_g}\right)}^2. $$

The overall discrepancy was computed as the weighted average

$$ \overline{\Omega}=\frac{\sum_{g=1}^G{N}_g{\Omega}_{g,t}}{N}, $$

where *t* is the maximum point in time of the last patients’ visits (*t* = 4 in the simulations). Larger values of $\overline{\Omega}$ reflect more severe violations of the normality assumption for the random effects.

We simulated 500 datasets for each combination of values of *N*, *n* and random-effects distribution parameters. For illustration purposes, selected BQQ plots are presented for *N* = 100 and *n* = 6 (Figs. 4, 5, 6, 7). These plots correspond to the datasets producing the $\overline{\Omega}$ closest to $\overline{\overline{\Omega}}$, where $\overline{\overline{\Omega}}$ is the average of the 500 values of $\overline{\Omega}$.

**Fig. 8:**
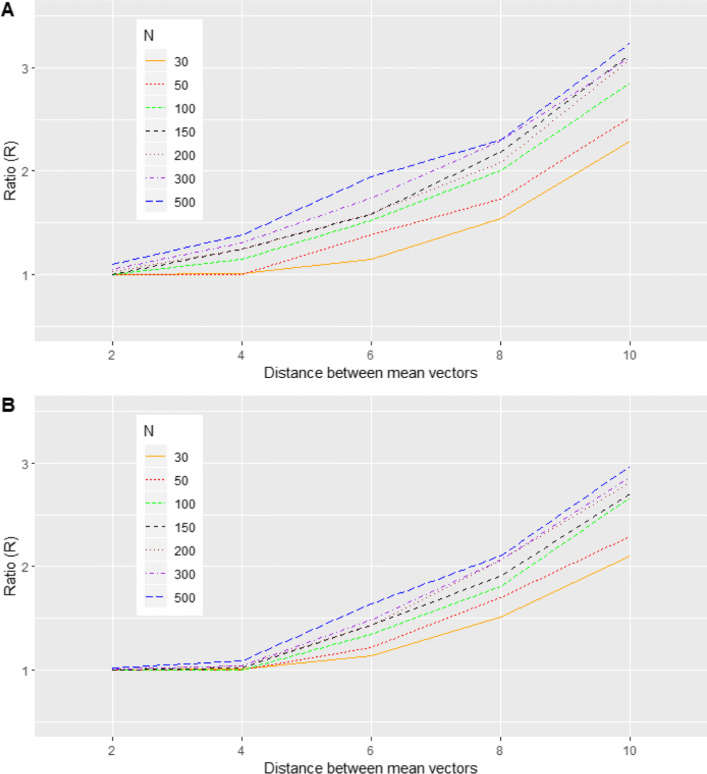
(Scenario 1). Ratios *R* comparing averages of CVM discrepancies under symmetric mixtures of two bivariate normal distributions versus comparable normal distributions with the same mean and variance-covariance matrix as a function of distance between means of the mixture components, for *N *= 30, 50, 100, 150, 200, 300 and 500. (**a**) *n* = 6. (**b**) *n* = 4

**Fig. 9:**
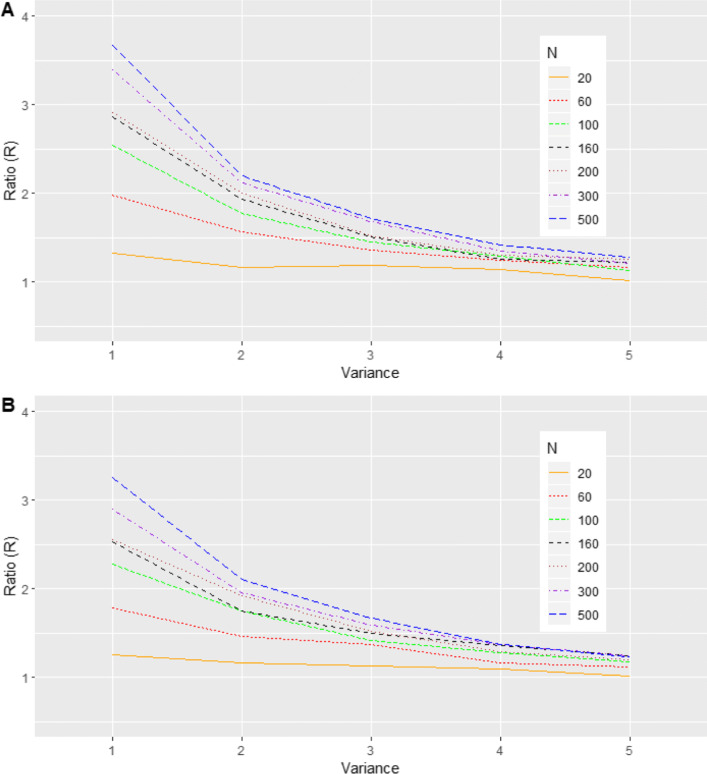
(Scenario 2). Ratios *R* comparing averages of CVM discrepancies under asymmetric mixtures of two bivariate normal distributions versus comparable normal distributions with the same mean and variance-covariance matrix as a function of the variance ${\sigma}_1^2={\sigma}_2^2$, for *N *= 20, 60, 100, 160, 200, 300 and 500. (**a**) *n* = 6. (**b**) *n* = 4

**Fig. 10:**
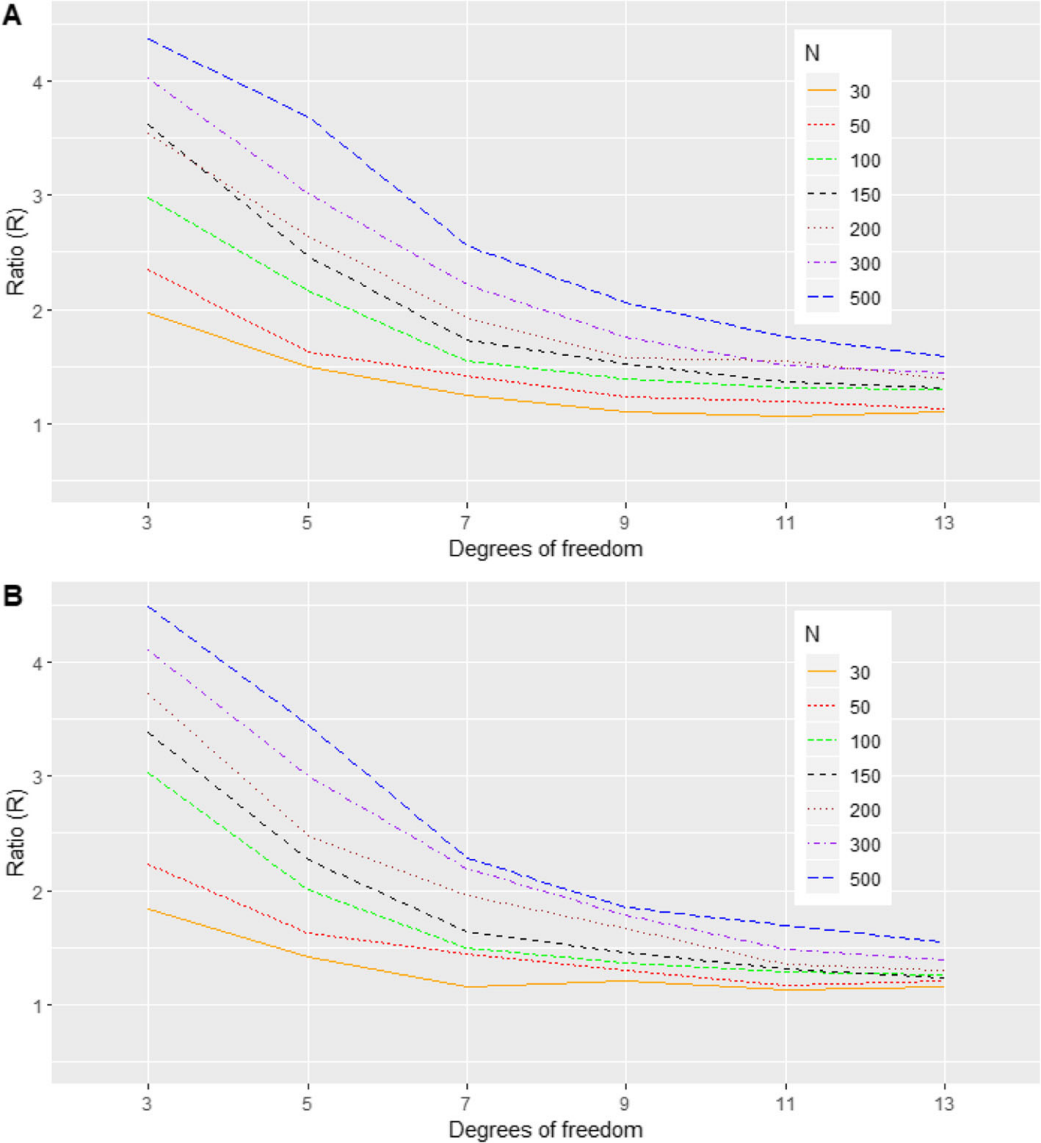
(Scenario 3). Ratios *R* comparing averages of CVM discrepancies under trivariate t distributions versus comparable normal distributions with the same mean and variance-covariance matrix as a function of the degrees of freedom *v*, for *N *= 30, 50, 100, 150, 200, 300 and 500. (**a**) *n* = 6. (**b**) *n* = 4

**Fig. 11:**
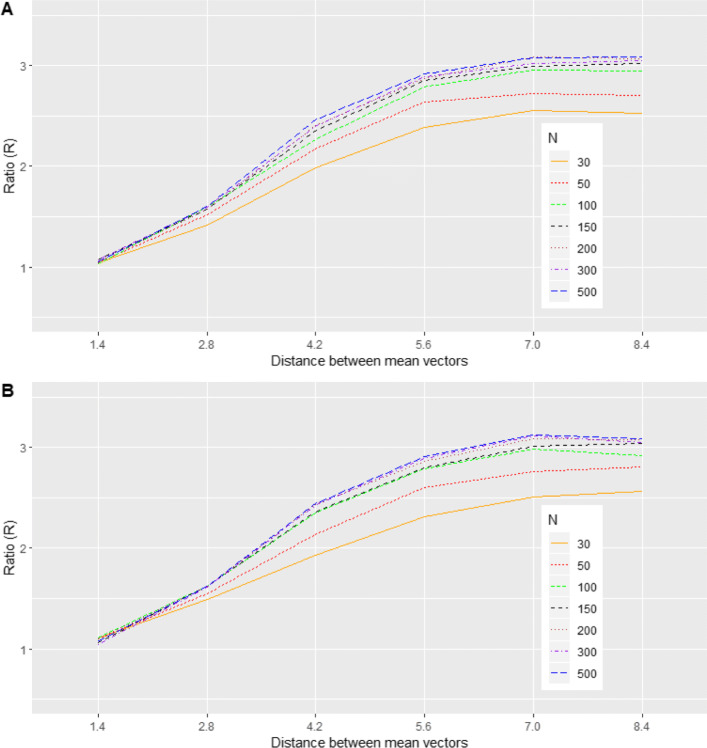
(Scenario 4). Ratios *R* comparing averages of CVM discrepancies under mixtures of two trivariate normal distributions versus comparable normal distributions with the same mean and variance-covariance matrix as a function of distance between means of the mixture components, for *N *= 30, 50, 100, 150, 200, 300 and 500. (**a**) *n* = 6. (**b**) *n* = 4

To examine the sensitivity of BQQ plots to detect deviations from normality, each simulated non-normal case was compared with its corresponding reference normal distribution by using the ratio $R=\frac{{\overline{\overline{\Omega}}}_{\mathrm{non}-\mathrm{normal}}}{{\overline{\overline{\Omega}}}_{\mathrm{normal}}}$ (Figs. 8, 9, 10, 11). On average, we expect the $\overline{\Omega}$ obtained from a non-normal case to be larger than that of its reference normal distribution and, therefore, *R* > 1. This is because Ω_*g*,*t*_ measures the discrepancy between the empirical distribution of the sample individual benefits and the theoretical distribution obtained under the normality assumption for the random effects. We expect larger values of *R* to be associated with greater deviations from normality. The SAS procedures MIXED and IML were used to implement the simulations (SAS Institute Inc. Cary, NC; see additional file SAS CODE.dat).

### Results of the simulation study

#### Scenario 1: symmetric mixtures of two bivariate normal distributions

As expected, larger distances between the two components of the mixture distribution determined more apparent asymmetric departures of the points on the BQQ plot from the *y = x* line (Fig. 4). By comparison, the BQQ plots for data simulated from the corresponding reference normal distributions tended not to show asymmetric deviations from the diagonal line. Figure 8 shows that the *R* ratios comparing CVM discrepancies of no-normal versus comparable normal distributions were always >1 and increased with the distance between the components of the mixture. In general, *R* increased with both the number of patients *N* and the number of repeated measures *n*, suggesting that the sample size contributes positively to the sensitivity of BQQ plots. Table S1 in the Supplementary Information shows the average CVM discrepancies $\overline{\overline{\Omega}}$ for all instances of Scenario 1.

#### Scenario 2: asymmetric mixtures of two bivariate normal distributions

For the investigated mixtures of normal distributions, quantiles of EB benefits from patient samples tended to be larger than the corresponding theoretical quantiles that assume normality for the random effects (Fig. 5). Moreover, this pattern was more apparent with smaller variances for the components of the mixture. The pattern was not observed in the BQQ plots corresponding to the reference normal distributions. Figure 9 shows that the ratios comparing CVM discrepancies of non-normal to reference normal distributions decreased with the variance of the mixture components, suggesting that BQQ plots are sensitive to deviations from normality. The ratios also increased with sample size *N* and the number of repeated measures *n*, suggesting that larger sample sizes increase the likelihood that BQQ plots capture normality violations. Table S2 in the Supplementary Information shows the average CVM discrepancies for the non-normal and normal cases.

#### Scenario 3: trivariate t distribution

As the degrees of freedom increased, the BQQ plots for data simulated with t distributions became more similar to the BQQ plots for data simulated with comparable normal distributions (Fig. 6). The theoretical quantiles of individual benefits under the normality assumption tended to be larger than the quantiles for EB sample benefits when the tails of the t distribution became heavier. Figure 10 shows that the ratios *R* comparing CVM discrepancies under t distributions versus their reference normal distributions increased as the degrees of freedom decreased, suggesting that BQQ plots can reliably capture the heaviness of the tails of the t distribution. The ratios tended to increase as both *N* and *n* increased, implying that the larger the sample size is the more efficient the proposed graphical approach is for detecting tail heaviness. Table S3 in the Supplementary Information shows average CVM discrepancies $\overline{\overline{\Omega}}$ for the non-normal and normal cases.

#### Scenario 4: symmetric mixture of two trivariate normal distributions

Analogous to scenario 1, marked departures in the appearance of BQQ plots from what is expected under comparable normal distributions are observed when the random effects are distributed as a mixture of normal distributions (Fig. 7). Greater distances between the two mean vectors of the mixture components tended to be associated with larger asymmetric deviations from the *y* = *x* line. This trend can also be inferred from Fig. 11, which shows that, compared with the reference normal distribution, CVM discrepancies under a mixture of normal distributions increased as the distance between the mixture components increased. Table S4 in Supplementary Information shows average CVM discrepancies for the non-normal and normal cases.

## Discussion

This article proposes a graphical approach to examining the normality assumption of the random effects of 2-PM models for severely ill patients. These models are based on RELMs and allow measuring individual benefits of medical or behavioral treatments [1, 2]. It is a common practice to explore the normality assumption for the random effects of RELMs by plotting separate classic normal QQ plots that examine the normality of the EB predictors of the random effects, for each random coefficient in the model [23, 24, 27, 46]. In the context of 2-PM models, however, using BQQ plots has two major advantages over normal QQ plots:

1. Whereas a goodness-of-fit analysis using normal QQ plots requires as many QQ plots as random coefficients are there in the model, only one BQQ plot is needed to examine the adequacy of the 2-PM model. Thus, BQQ plots reduce the number of goodness-of-fit analyses if the model has two or more random coefficients.
2. In contrast with normal QQ plots, BQQ plots examine directly the predictors of the individual benefits achieved by the patient sample. Therefore, BQQ plots contribute to assess whether the 2-PM model is appropriate for predicting individual benefits, which is the model’s main purpose. In this regard, note that BQQ plots may not be useful if the goal of the RELM is not to measure individual benefits of medical treatments. In fact, as described above, the shape of a BQQ plot depends on the therapeutic target, which is prespecified in advance by the clinician. Also note that if the 2-PM model will be used for measuring individual benefits in new patients, the EB predictors of individual benefits need to be additionally evaluated using the simulation approach described by Diaz [1].

An essential difference between normal QQ plots and BQQ plots is that whereas normal QQ plots represent visually the deviation of an empirical distribution from an estimated normal distribution, BQQ plots represent the deviation of an empirical distribution from an estimate of a non-normal distribution. This non-normal distribution is the one given by Eq. (2). This feature of BQQ plots is the reason we chose the CVM discrepancy for evaluating the performance of BQQ plots over the more common Shapiro-Wilk test statistic [43, 44, 47]. In fact, although the Shapiro-Wilk statistic has been shown to be more sensitive to deviations from linearity in normal QQ plots than the CVM and other discrepancy measures [48], it measures deviations from linearity by explicitly using the fact that the theoretical quantiles in the x axis of normal QQ plots are those of a normal distribution [41]. Therefore, Shapiro-Wilk statistic cannot be used to measure departures from linearity in BQQ plots. In contrast, the CVM discrepancy is a direct measure of the discrepancy between the two distributions that are being compared in BQQ plots: the empirical cumulative distribution function of the EB predictors of individual benefits of the patient sample versus an estimate of the non-normal cumulative distribution function of individual benefits. There are other measures that can potentially be used to assess discrepancies between these two distributions; for instance, the Kolmogorov-Smirnov distance and the Anderson-Darling test statistic [42, 45, 48, 49]. However, by using CVM discrepancies, we were able to show in the current study that BQQ plots are sensitive to deviations from the normality assumption of the random effects (Figs. 4, 5, 6, 7, 8, 9, 10, 11).

In addition to the aforementioned limitations of using classic normal QQ plots that examine the normality of the EB predictors of random effects to assess 2-PM models, note that, in normal QQ plots, the EB predictors are used to compute *both* the sample quantiles represented in the y axis and the mean and variance of the normal distribution used to obtain the theoretical quantiles represented in the x axis. This circularity limits the interpretation of the resultant plot because the EB predictors are estimates themselves. Moreover, research shows that the shape of the empirical distribution of the EB predictors of the random effects does not necessarily reflect the shape of the random-effects distribution and tends to be unimodal [16, 19], which may make the plot look artificially linear. Thus, QQ plots calculated with only EB predictors of the random effects may be misleading as a tool for examining the normality assumption. In contrast, in our proposed approach, the theoretical quantiles given in Eq. (3) are estimated directly using the MLEs or RMLEs of model parameters without the mediation of the EB predictors of random effects or the EB predictors of individual benefits. In addition, our simulations show that BQQ plots reliably perform as expected under non-normal and normal distributions. Thus, if the model will be used to make decisions related with personalized medicine, we recommend using BQQ plots as a complementary tool for the exploration of the normality assumption for the random effects.

When the BQQ plot suggests that the assumption of normality for the random effects is not reasonably valid, data modelers can utilize linear mixed models that assume mixtures of normals [19], multivariate t distributions [50], multivariate Laplace distributions [51], or skew-normal distributions [52]. Individual benefits can still be predicted by plugging the estimates of the fixed effects and variance components as well as the EB predictors of random effects into Eq. (2). Of these models, those with mixtures of normals or skew-normal distributions are particularly attractive since they have closed-form formulas for the EB predictors of the random effects that may facilitate the measurement of individual benefits in new patients [1]. Note that EB predictors of random effects in these models no longer inherit the robustness properties of the BLUPs since they are distribution dependent. Research investigating the accuracy of prospective and retrospective measures of individual benefits in new patients based on these predictors is needed [1].

QQ plots are widely used in statistical practice to examine distributional assumptions for a variety of models, not necessarily models that assume normality [24, 27, 41, 53–56]. A common criticism of these plots is that they are less objective than classical goodness-of-fit tests. However, psychophysical studies have shown that QQ plots, when visually examined in comparison with plots simulated under the null distribution, are more powerful for detecting deviations from the null distribution than classical tests [40, 41, 56]. This is probably because in QQ plots the entire sample is assessed rather than a single test statistic [41]. We believe that this is a strong reason for using BQQ plots in addition to analyses based on classical goodness-of-fit tests. Another reason is that classical tests are frequently uninformative when the sample size is very small or very large [57]. It is well known, for instance, that powerful normality tests conducted with sufficiently large samples reject firmly the hypothesis of normality when the deviation from normality is trivial. This is a limitation because useful real-world applications of statistical models assuming normality need only approximate normality, not perfect normality. On the other hand, if a statistical test rejects the null hypothesis of perfect goodness-of-fit, a QQ plot allows assessing why there is a lack of fit and whether the lack of fit is negligible compared to plots simulated under the null hypothesis [41]. QQ plots also help in the selection of appropriate transformations of the response or transformations of covariates for model improvement.

Here, we compared the BQQ plot computed with the real data with plots computed with simulated data that assume normality for the random effects in order to gauge more objectively the deviation of the plot from the y = x line (Fig. 3). To further prevent subjectivity in the interpretation of BQQ plots, formal lineup statistical tests may be conducted [40, 41, 56]. In these tests, the BQQ plot for the real data would be lined up randomly and blindly with several BQQ plots computed with simulated data consistent with the null hypothesis of normality for the random effects, and the data analyst would attempt to single out the plot for the real data from the other plots. Formal inferential procedures for computing *p*-values for these types of tests have been proposed [40, 41, 56]. Lineup tests, however, require that the analyst does not see the plot for the real data before conducting the test to prevent biases from preconceived conclusions or psychophysical artifacts. Further research is needed to formally compare the statistical power of lineup tests for BQQ plots with the power of statistical tests of normality for random effects linear models based on regular test statistics [32, 34, 35, 58, 59]. Due to the graphical nature of BQQ plots, such comparison requires the conduction of graphical perception experiments in human subjects that implement methods of visual statistical inference [40, 41, 56].

To illustrate our proposed graphical approach, we used data from 66 subjects of an imipramine clinical trial [6, 39]. As discussed by Diaz [1], this sample size was the result of a careful application of strict inclusion and exclusion criteria that ensured that the subjects were at a stable illness state before entering the study. The inclusion criteria included three numerical constraints on the individual items of the HRS that reflected severity of the depression. Eighteen subjects who did not satisfy these constraints at the end of the placebo period were excluded from the study. In addition, subjects who experienced clinically relevant disease or procedural changes during the baseline period or later were also excluded (for instance, electroconvulsive therapy, suicide attempt or evolution to mania [39]). A total of 36 subjects were excluded. This rigorous approach ensured that baseline disease severity was a reliable scientific construct and that the observed reductions in disease severity during imipramine treatment were not the result of the natural history of the disease.

Our simulations showed that the sensitivity of BQQ plots for detecting non-normality increased with the sample size. They also showed that moderate sample sizes (50 ≤ *N* ≤ 100), which are common in clinical trials, were enough to detect deviations from normality in many of the investigated cases, as suggested by Figs. 8, 9, 10, 11. Moreover, with smaller sample sizes (*N* = 30), BQQ plots exhibited some sensitivity to non-normality under symmetric mixtures of normal distributions, which may occur if there are omitted categorical variables [19] (Figs. 8 and 11).

A limitation of our approach is that it requires sufficiently large *N*_*g*_ values to guarantee that sample quantiles are sufficiently close to theoretical quantiles. Moreover, the approach is not applicable when *N*_*g*_ = 1 for some *g*, which may occur if the model has a relatively high number of covariates. Future research must investigate how to overcome these limitations. A possible solution is to treat ***X*** as a stochastic covariate vector and to work with the marginal cumulative distribution function of the individual benefits *F*_*m*_(*z*) = *F*_*m*_(*z*; *t*) = *E*[*F*(*z*; ***X***, *t*)], where the expected value is taken with respect to the joint probability mass function of ***X*.** A reasonable estimate of *F*_*m*_(*z*) is ${\hat{F}}_m(z)={N}^{-1}{\sum}_{g=1}^G{N}_g\hat{F}\left(z;{\boldsymbol{x}}_g,t\right)$, where $\hat{F}$ is obtained by replacing fixed effects and variance components in Eq. (2) with their corresponding estimates. An estimate of the marginal *p*-th quantile function is, therefore, ${\hat{B}}_m(p)={\hat{B}}_m\left(p;t\right)={\hat{F}}_m^{-1}(p)$, which can be obtained through numerical inversion. Thus, the BQQ plot can be computed as the points $\left({\hat{B}}_m\left(\frac{i-0.5}{N};t\right),{\hat{b}}_{t,(i)}\right)$, *i* = 1, …, *N*, where ${\hat{b}}_{t,(1)}<{\hat{b}}_{t,(2)}<\dots <{\hat{b}}_{t,(N)}$ are the order statistics of the combined sample of EB-predicted individual benefits. Future research must examine the extent to which this plot is sensitive to deviations from normality.

Another limitation of our approach is that it requires that continuous or ordinal covariates be categorized before implementing Eq. (3). Future research must examine how to incorporate continuous covariates to BQQ plots. The extension of BQQ plots to 2-PM models with non-continuous responses also needs further research. The influence of missing observations on the performance of BQQ plots must also be investigated.

Diaz [1] showed that when the patients are severely ill, a closed-form formula for the probability distribution of individual benefits can be obtained. This facilitates both the statistical analyses of individual benefits and the evaluation of the overall performance of the predictors of individual benefits in new patients. Some clinical studies, however, may include patients who are not severely ill. Further research must investigate how to extend the definition of BQQ plots to these other studies. Since there is no closed-form formula in this case, additional numerical or Monte Carlo integration may be needed.

## Conclusions

This paper proposes a graphical approach to evaluate the goodness-of-fit of random effects models for continuous responses when the purpose of the model is to estimate individual benefits of medical treatments. In our approach, empirical quantiles of individual benefits estimated with an empirical Bayes approach are plotted against the quantiles of the distribution of individual benefits calculated under a normality assumption for the random effects. The rationale underlying our approach is that EB predictors of the random effects are robust to violations of the normality assumption [15–17]. In fact, EB predictors are also estimates of the BLUPs, whose optimality properties do not require the normality assumption [13].

If the normality assumption is valid, we expect empirical quantiles to be close to the theoretical quantiles. Thus, we can infer the goodness-of-fit of the 2-PM model if the BQQ plot does not show obvious asymmetric deviations from the *y* = *x* diagonal line. We evaluated the performance of this approach using the CVM discrepancy which measures the discrepancy between an empirical and a theoretical probability distribution. CVM discrepancies confirmed that our graphical approach captures accurately deviations from the normality assumption. Importantly, we found that the ratios *R* of average CVM discrepancies ($\overline{\overline{\Omega}}$), which compared non-normal distributions with closely comparable normal distributions, were not smaller than 1 in all simulations. This suggests that BQQ plots are powerful tools for detecting deviations from normality of the distribution of the random effects in 2-PM models and for a visual confirmation of the normality assumption as well, when the goal is to evaluate the ability of the model to gauge individual treatment benefits.

## Supplementary information

**Additional file 1.** Supplementary Information. Additional figures and tables.

**Additional file 2.** SAS/IML code. Computer code implementing the graphical approach and calculating Cramer-von Mises discrepancies.

## Data Availability

The depression trial data are available from the corresponding author upon request. The computer code for this paper is available as an additional file and simulated datasets are available from the corresponding author on request.

## Abbreviations

2-PM: 2-dimensional personalized medicine
BLUP: Best linear unbiased predictor
BQQ: Benefit quantile-quantile
CVM: Cramer-von Mises
EB: Empirical Bayes
HRS: Hamilton Rating Scale
MLE: Maximum likelihood estimator
REML: Restricted maximum likelihood estimator
RELM: Random-effects linear model
QQ: Quantile-quantile
