# Supplementary material for: A graphical approach to assess the goodness-of-fit of random-effects linear models when the goal is to measure individual benefits of medical treatments in severely ill patients
Source: BMC Med Res Methodol. 2020 Jul 20;20:193. doi: 10.1186/s12874-020-01054-3 (PMC7370523; doi:10.1186/s12874-020-01054-3)
Supplement: Supplementary file 1 — Additional file 1. Supplementary Information. Additional figures and tables. [file 12874_2020_1054_MOESM1_ESM.pdf]

Supplementary Information

A Graphical Approach to Assess the Goodness-of-Fit of Random Effects Linear Models When the Goal is to Measure Individual Benefits of Medical Treatments in Severely Ill Patients

Zhiwen Wang[1] and Francisco J. Diaz[1,*]

[1]Department of Biostatistics, The University of Kansas Medical Center, Mail Stop 1026, 3901 Rainbow Blvd., Kansas City, KS 66160, United States.

*Correspondence: fdiaz@kumc.edu.

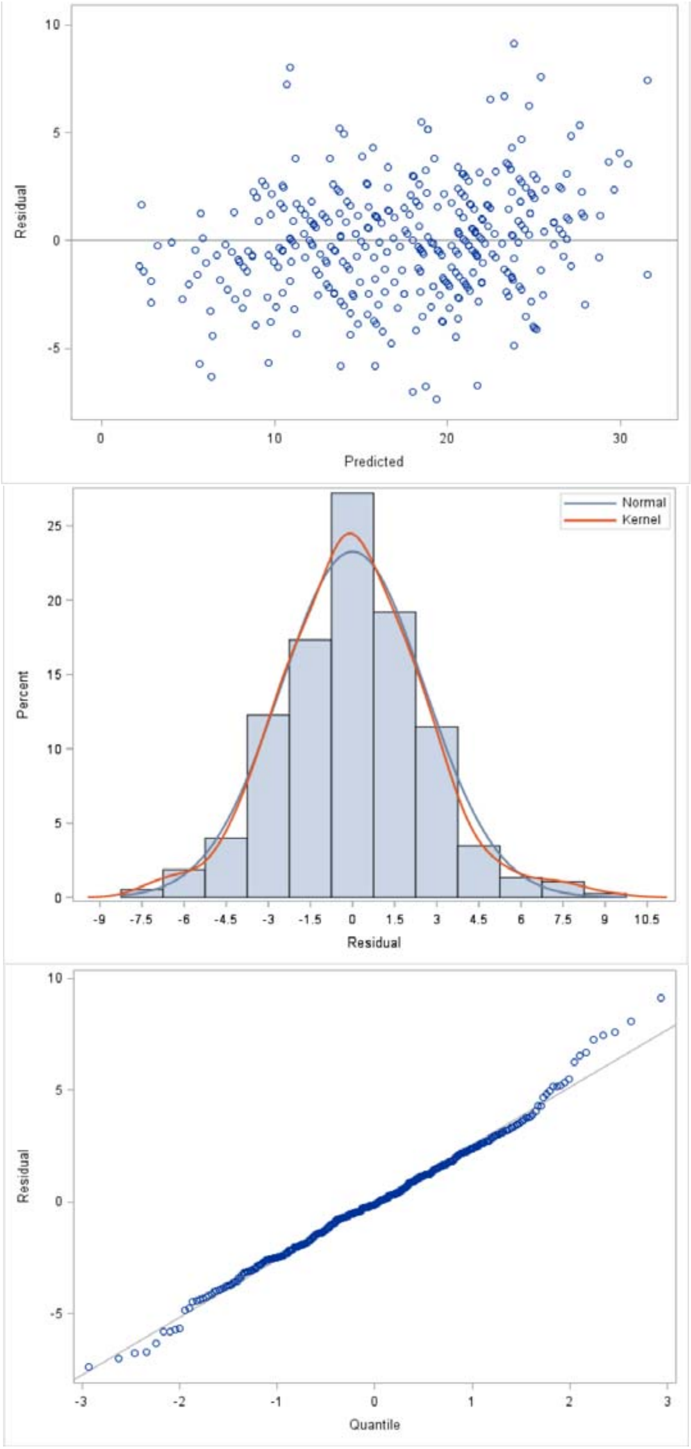

Figure S1. Scatterplot, histogram and normal Q-Q plot of conditional residuals for the random effects linear model of the depression data.

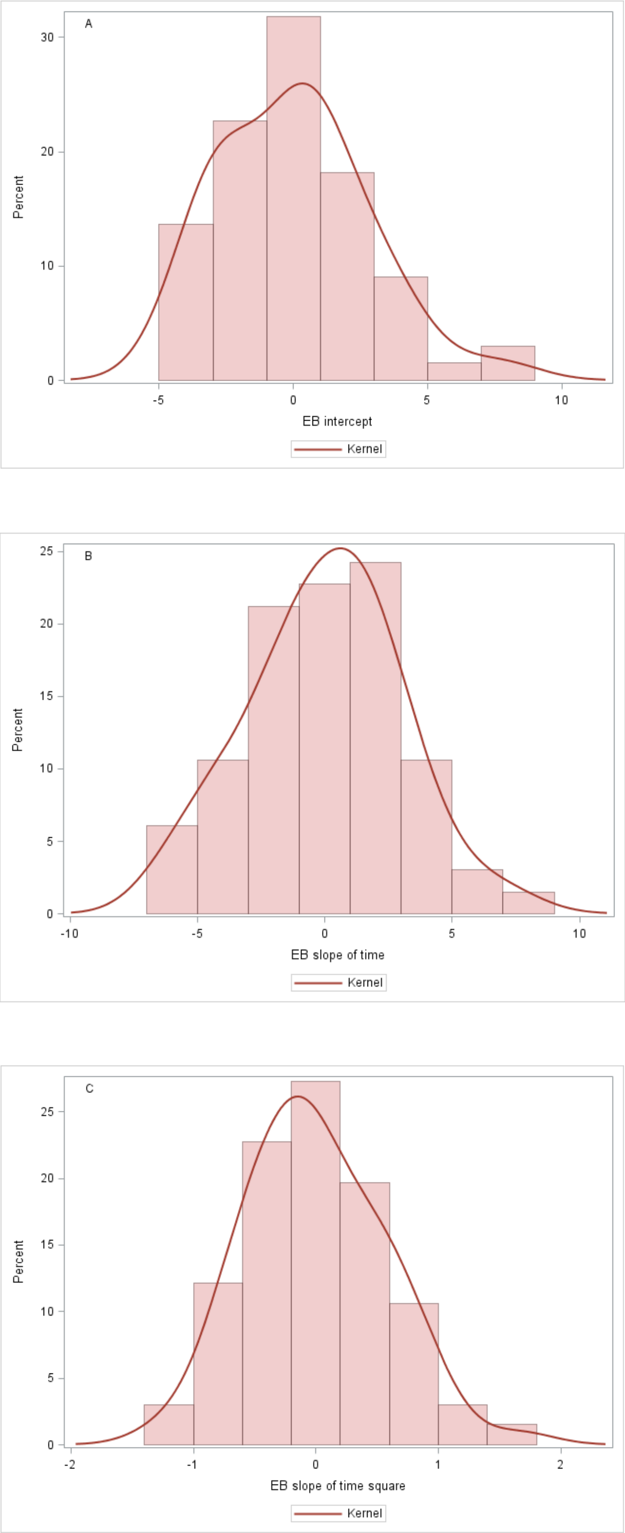

Figure S2. Histograms and kernel densities of empirical Bayes (EB) predictors of the random effects for 66 patients with depression under imipramine treatment. (A) Predictors of the Random

intercept. (B) Predictors of the random slope for time. (C) Predictors of the random slope for time square.

Table S1 (Scenario 1). Average Cramer-von Mises discrepancies $\bar{\bar{\Omega}}$ between the empirical distribution of EB predicted individual benefits and the theoretical distribution of benefits that assumes normality for the random effects, for simulated reference bivariate normal (RBN) distributions and symmetric mixtures of two bivariate normal distributions with the same mean and variance-covariance matrix, by distance between mean vectors of the mixture components.

$n = 6$	Distance between means									
	2		4		6		8		10	
N	RBN	Mixture	RBN	Mixture	RBN	Mixture	RBN	Mixture	RBN	Mixture
30	0.00703	0.00703	0.00872	0.00879	0.0127	0.0146	0.0188	0.0290	0.0238	0.0544
50	0.00434	0.00435	0.00552	0.00555	0.00818	0.0113	0.0135	0.0233	0.0189	0.0475
100	0.00216	0.00216	0.00300	0.00343	0.00530	0.00807	0.00990	0.0198	0.0147	0.0418
150	0.00137	0.00138	0.00197	0.00246	0.00434	0.00686	0.00847	0.0185	0.0132	0.0414
200	0.00113	0.00116	0.00172	0.00215	0.00408	0.00648	0.00828	0.0173	0.0129	0.0398
300	0.000711	0.000746	0.00127	0.00165	0.00348	0.00604	0.00735	0.0168	0.0125	0.0386
500	0.000527	0.000579	0.000962	0.00133	0.00298	0.00579	0.00701	0.0161	0.0118	0.0381

$n = 4$	Distance between means									
	2		4		6		8		10	
N	RBN	Mixture	RBN	Mixture	RBN	Mixture	RBN	Mixture	RBN	Mixture
30	0.00722	0.00722	0.00881	0.00887	0.0128	0.0146	0.0186	0.0281	0.0252	0.0530
50	0.00455	0.00455	0.00530	0.00532	0.00870	0.0106	0.0140	0.0238	0.0194	0.0445
100	0.00245	0.00245	0.00290	0.00290	0.00566	0.00761	0.0101	0.0182	0.0149	0.0396
150	0.00168	0.00168	0.00218	0.00222	0.00452	0.00646	0.00895	0.0170	0.0135	0.0364
200	0.00133	0.00135	0.00169	0.00175	0.00419	0.00599	0.00797	0.0164	0.0127	0.0358
300	0.00100	0.00102	0.00135	0.00140	0.00365	0.00540	0.00761	0.0157	0.0123	0.0353
500	0.000713	0.000730	0.00100	0.00109	0.00312	0.00510	0.00707	0.0149	0.0118	0.0352

Table S2 (Scenario 2). Average Cramer-von Mises discrepancies $\bar{\bar{\Omega}}$ between the empirical distribution of EB predicted individual benefits and the theoretical distribution of benefits that assumes normality for the random effects, for simulated reference bivariate normal (RBN) distributions and mixtures of two bivariate normal distributions with the same mean and variance-covariance matrix and selected variances in the mixture components $\sigma_1^2 = \sigma_2^2$.

$n = 6$	σ_1^2, σ_2^2									
	1		2		3		4		5	
N	RBN	Mixture	RBN	Mixture	RBN	Mixture	RBN	Mixture	RBN	Mixture
20	0.0113	0.0150	0.0134	0.0157	0.0135	0.0161	0.0148	0.0169	0.0161	0.0163
60	0.00660	0.0131	0.00808	0.0127	0.00932	0.0126	0.0110	0.0136	0.0119	0.0138
100	0.00484	0.0123	0.00621	0.0110	0.00746	0.0108	0.00886	0.0114	0.0104	0.0118
160	0.00373	0.0107	0.00498	0.00965	0.00622	0.00941	0.00793	0.00992	0.00887	0.0108
200	0.00352	0.0103	0.00468	0.00936	0.00610	0.00931	0.00730	0.00947	0.00827	0.0104
300	0.00289	0.00982	0.00424	0.00897	0.00541	0.00909	0.00678	0.00917	0.00789	0.00955
500	0.00261	0.00958	0.00384	0.00846	0.00494	0.00849	0.00611	0.00865	0.00733	0.00939

$n = 4$	σ_1^2, σ_2^2									
	1		2		3		4		5	
N	RBN	Mixture	RBN	Mixture	RBN	Mixture	RBN	Mixture	RBN	Mixture
20	0.0120	0.0151	0.0139	0.0162	0.0144	0.0163	0.0157	0.0173	0.0176	0.0178
60	0.00707	0.0126	0.00814	0.0119	0.00914	0.0125	0.0114	0.0132	0.0124	0.0139
100	0.00473	0.0108	0.00600	0.0105	0.00753	0.0106	0.00859	0.0110	0.0103	0.0121
160	0.00374	0.00946	0.00521	0.00911	0.00617	0.00927	0.00747	0.0102	0.00879	0.0110
200	0.00356	0.00910	0.00466	0.00896	0.00592	0.00902	0.00729	0.00940	0.00839	0.0101
300	0.00303	0.00877	0.00423	0.00828	0.00539	0.00854	0.00657	0.00903	0.00768	0.00957
500	0.00257	0.00839	0.00380	0.00801	0.00491	0.00822	0.00613	0.00841	0.00746	0.00918

Table S3 (Scenario 3). Average Cramer-von Mises discrepancies $\bar{\bar{\Omega}}$ between the empirical distribution of EB predicted individual benefits and the theoretical distribution of benefits that assumes normality for the random effects, for simulated reference trivariate normal (RTN) distributions and trivariate t distributions with the same mean and variance-covariance matrix, by degrees of freedom v.

$n = 6$	Degrees of freedom (v)											
	3		5		7		9		11		13	
N	RTN	t	RTN	t	RTN	t	RTN	t	RTN	t	RTN	t
30	0.0123	0.0241	0.00857	0.0128	0.00800	0.0100	0.00798	0.00884	0.00768	0.00820	0.00748	0.00823
50	0.00842	0.0197	0.00528	0.00859	0.00473	0.00671	0.00469	0.00579	0.00460	0.00548	0.00446	0.00505
100	0.00567	0.0169	0.00288	0.00626	0.00268	0.00416	0.00248	0.00347	0.00245	0.00322	0.00235	0.00306
150	0.00463	0.0168	0.00219	0.00541	0.00190	0.00328	0.00174	0.00263	0.00170	0.00232	0.00165	0.00216
200	0.00436	0.0155	0.00182	0.00480	0.00149	0.00286	0.00142	0.00224	0.00131	0.00204	0.00129	0.00178
300	0.00384	0.0155	0.00143	0.00432	0.00117	0.00259	0.00107	0.00189	0.00103	0.00155	0.000996	0.00143
500	0.00345	0.0151	0.00108	0.00400	0.000833	0.00213	0.000768	0.00158	0.000728	0.00128	0.000685	0.00109

$n = 4$	Degrees of freedom (v)											
	3		5		7		9		11		13	
N	RTN	t	RTN	t	RTN	t	RTN	t	RTN	t	RTN	t
30	0.0131	0.0239	0.00886	0.0125	0.00870	0.0100	0.00805	0.00969	0.00804	0.00904	0.00765	0.00885
50	0.00888	0.0198	0.00551	0.00897	0.00489	0.00704	0.00471	0.00611	0.00471	0.00551	0.00456	0.00553
100	0.00557	0.0168	0.00316	0.00635	0.00276	0.00412	0.00257	0.00350	0.00249	0.00320	0.00244	0.00308
150	0.00479	0.0162	0.00232	0.00527	0.00204	0.00335	0.00179	0.00261	0.00189	0.00249	0.00179	0.00222
200	0.00419	0.0156	0.00191	0.00474	0.00158	0.00307	0.00145	0.00242	0.00147	0.00199	0.00143	0.00186
300	0.00381	0.0157	0.00147	0.00442	0.00121	0.00266	0.00109	0.00194	0.00112	0.00167	0.00105	0.00147
500	0.00342	0.0154	0.00117	0.00405	0.000952	0.00217	0.000847	0.00157	0.000816	0.00138	0.000789	0.00122

Table S4 (Scenario 4). Average Cramer-von Mises discrepancies $\bar{\bar{\Omega}}$ between the empirical distribution of EB predicted individual benefits and the theoretical distribution of benefits that assumes normality for the random effects, for simulated reference trivariate normal (RTN) distributions and mixtures of two trivariate normal distributions with the same mean and variance-covariance matrix, by distance between mean vectors of the mixture components.

$n = 6$	Distance between means											
	1.4		2.8		4.2		5.6		7.0		8.4	
N	RTN	Mixture	RTN	Mixture	RTN	Mixture	RTN	Mixture	RTN	Mixture	RTN	Mixture
30	0.0344	0.0358	0.0505	0.0714	0.0601	0.119	0.0627	0.150	0.0646	0.165	0.0656	0.166
50	0.0282	0.0295	0.0439	0.0665	0.0528	0.115	0.0563	0.149	0.0582	0.158	0.0590	0.160
100	0.0246	0.0253	0.0394	0.0626	0.0502	0.114	0.0525	0.146	0.0530	0.157	0.0536	0.158
150	0.0233	0.0250	0.0386	0.0608	0.0484	0.114	0.0507	0.144	0.0521	0.156	0.0517	0.156
200	0.0228	0.0239	0.0388	0.0611	0.0471	0.113	0.0504	0.145	0.0505	0.156	0.0513	0.157
300	0.0222	0.0237	0.0379	0.0603	0.0466	0.112	0.0498	0.144	0.0512	0.155	0.0510	0.156
500	0.0221	0.0233	0.0374	0.0600	0.0459	0.113	0.0495	0.145	0.0505	0.155	0.0504	0.156

$n = 4$	Distance between means											
	1.4		2.8		4.2		5.6		7.0		8.4	
N	RTN	Mixture	RTN	Mixture	RTN	Mixture	RTN	Mixture	RTN	Mixture	RTN	Mixture
30	0.0383	0.0424	0.0508	0.0756	0.0627	0.121	0.0662	0.153	0.0640	0.161	0.0650	0.167
50	0.0303	0.0333	0.0442	0.0686	0.0556	0.119	0.0569	0.148	0.0575	0.159	0.0576	0.162
100	0.0255	0.0281	0.0398	0.0645	0.0495	0.116	0.0525	0.147	0.0527	0.157	0.0540	0.158
150	0.0243	0.0258	0.0394	0.0638	0.0484	0.114	0.0519	0.145	0.0520	0.157	0.0522	0.159
200	0.0222	0.0243	0.0378	0.0613	0.0470	0.114	0.0508	0.145	0.0506	0.156	0.0512	0.157
300	0.0228	0.0237	0.0379	0.0610	0.0471	0.114	0.0502	0.145	0.0502	0.156	0.0512	0.156
500	0.0215	0.0231	0.0375	0.0608	0.0466	0.114	0.0498	0.145	0.0500	0.156	0.0509	0.157
